# A data-driven approach to manage type 2 diabetes mellitus through digital health: The Klivo Intervention Program protocol (KIPDM)

**DOI:** 10.1371/journal.pone.0281844

**Published:** 2023-02-24

**Authors:** Camila Maciel de Oliveira, Luiza Borcony Bolognese, Mercedes Balcells, Davi Casale Aragon, Roberto Luis Zagury, Clemente Nobrega, Chunyu Liu

**Affiliations:** 1 Klivo LLC, São Paulo, Brazil; 2 Health Innovation Program, Medical School, The Pontifical Catholic University of Minas Gerais, Poços de Caldas, Brazil; 3 Institute for Medical Engineering and Science, Massachusetts Institute of Technology, Cambridge, MA, United States of America; 4 University of São Paulo, Ribeirão Preto, Brazil; 5 State Institute of Diabetes and Endocrinology Luiz Capriglione (IEDE), Rio de Janeiro, Brazil; 6 Framingham Heart Study, Framingham, MA, United States of America; 7 Department of Biostatistics, Boston University, Boston, MA, United States of America; Centre Hospitalier Sud Francilien, FRANCE

## Abstract

**Background:**

Digital therapeutics, an emerging type of medical approach, is defined as evidence-based therapeutic interventions through qualified software programs that help prevent, manage, or treat chronic diseases such as type 2 diabetes mellitus (T2DM), which has high social and economic burden. Klivo, a startup certified by the Brazilian Society of Diabetes, developed the first digital therapeutic product for managing T2DM in Brazil, reaching 21 of 24 states. Klivo has continuously been improving its model of behavior change on the basis of an intensive lifestyle intervention method that addresses individuals’ needs–the Klivo Intervention Program for T2DM (KIPDM). To test the most recent version of the KIPDM, we will evaluate the ongoing management of daily life habits in patients with T2DM by measuring clinically significant outcomes. To improve the transparency of further results, here we will present the study protocol and detail the plan for the research project, including the study design and the analysis strategies.

**Methods:**

The KIPDM will be sponsored by health plans and healthcare provider organizations and will be free for patients (adults aged ≥ 18 years and <65 years; and glycated hemoglobin ≥ 7%). The program will be based on a 6-month management process that will supervise patients remotely. The program will include educational classes via the Klivo app, text messages, or e-mails. Evaluation will include objectively assessing clinical, laboratory, and behavioral outcomes such as health-related quality of life, mental health, medication adherence, and healthcare utilization. For this, validated electronic questionnaires will be available through the Klivo app. The primary outcome will be glycated hemoglobin (HbA1c) values. The secondary outcome will be time in target blood glucose range (TIR) estimated by capillary glycemia. Other outcomes of interest will be evaluated at baseline and stipulated time points (3 and 6 months after the start of the program).

**Expected outcomes:**

KIPDM patients should present improved HbA1c and TIR along the intervention as compared to baseline values. Findings from this study will provide insights into the health improvement of T2DM and other cardiometabolic conditions such as hypertension, dyslipidemia, and obesity by using a digital therapeutic strategy. By analyzing the patient’s health over time, this study will also contribute to understanding comorbidities associated with this chronic condition in the Brazilian population.

## Introduction

Digital therapeutics–an emerging type of medical approach that is expanding globally with market demand–is defined as evidence-based therapeutic interventions through qualified software programs that help prevent, manage, and treat chronic diseases [1]. Some studies have suggested that continuous remote evaluation and daily monitoring can effectively refine the management of chronic conditions such as type 2 diabetes mellitus (T2DM), which has a high prevalence and social and economic burden [2, 3]. In this sense, digital therapeutics products addressed to patients’ individual needs have contributed to ongoing management of daily life habits and to lowering healthcare costs attributed to chronic metabolic diseases [4, 5].

These technologies have improved diet and exercise, optimized glycemic control, and ensured adherence to medication use, consequently lowering the high cost of treating T2DM [6]. Indeed, clinical advancement of patients participating in protocols such as the Livongo for Diabetes Program has lowered average costs per patient per month by $83 [7], which is crucial if we consider the estimation that 700 million people will be living with diabetes in 2045 in the world [2]. Also, programs that support lifestyle changes in patients with T2DM can perform secondary prevention by reducing the risk of other chronic complications [8].

Nowadays, there is a consensus that value is created by enabling health and not by just delivering care. In this scenario, some healthcare organizations have offered digital programs as part of the value-based care model [9]. Furthermore, people who use digital health technologies benefit from short-term health improvements and long-term health self-management [10]. Therefore, here we intend to summarize the use of a digital health strategy for managing T2DM–the Klivo Intervention Program (KIPDM)–and to present the study protocol detailing the plan for the research project, including the study design and analysis strategies.

## Methods

### Overview

Klivo is a Brazilian startup founded in 2020 and certified by the Brazilian Society of Diabetes in February 2022. This group has developed the first digital therapeutic strategy in this country for managing T2DM by addressing individuals’ needs and targeting adults with HbA1c of 7% or higher.

The Klivo app was created in the Portuguese language, and this program has been continuously improved during the test stage (2020 to 2022). This model of behavior change based on an intensive lifestyle intervention method was then called the Klivo Intervention Program for T2DM (KIPDM).

After a preliminary retrospective analysis, the methodology was adjusted for improvements and will run digital, but phone calls will be made if an emergency such as hypo- or hyperglycemia occurs. Therefore, this study protocol is a 6-month design that will involve testing the adjusted behavior intervention while gathering information on implementation strategies and outcomes. Clinical, laboratory, and behavioral aspects will be evaluated for the patients recruited for this cohort. Additionally, mental health, medication adherence, and healthcare utilization will be evaluated by using validated questionnaires. This design will also evaluate metrics of program delivery, fidelity, and processes that can affect program success. The patients will input their data in the Klivo app.

Glycated hemoglobin (HbA1c) will be considered as the primary outcome. The secondary outcome will be time in range (TIR), defined by the percentage of time the patients spend with their blood glucose levels in a target range of 70 to 180 mg/dL. Other outcomes will be described below.

### Study objectives

#### Primary outcome

The goal is to evaluate glycated hemoglobin (HbA1c) values at baseline (before the intervention starts) and 3 and 6 months after the intervention starts. A decrease in HbA1c, measured as absolute point, is expected. HbA1c will be evaluated by laboratory exams requested by the patient’s medical doctor and input by the patient via the Klivo app.

A 0.4% decrease in HbA1c post-intervention, every three months, will be considered a successful outcome. The goal is a minimum Hb1Ac value of 7%. When this value is reached, the goal will be to maintain this value and not to reduce it even further [11].

#### Secondary outcome

To evaluate change in time in the target blood glucose range (TIR) values at 3 and 6 months after the intervention starts will be compared to TIR at baseline (before the intervention starts). The number of measurements within the ideal blood glucose range is expected to increase along time. The number of hypoglycemic (< 70 mg/dL) events over 6 months will also be verified.

Although Continuous Glucose Monitoring (CGM) is the gold standard for estimating TIR, capillary glycemia will be used in this protocol. Capillary glucose, in mg/dL, will be measured with a glucometer, which will be sent to the patient’s home address. The glucose level will be obtained with the Klivo app, downloaded on the patient’s smartphone, connected to the glucometer. At least a 5% increase in TIR every three months will be considered a successful outcome.

#### Other outcome measures

The KIPDM is expected to impact the following characteristics positively at 3 and 6 months after the intervention starts compared to baseline (month 1): clinical (weight, percentage of body weight loss, blood pressure, waist circumference), laboratory exams (total cholesterol, HDL-c, LDL-c, triglycerides, creatinine), and others (presence of retinal, renal, cardiac, and cerebrovascular complications; health-related quality of life, medication adherence, and mental health status; and health care utilization).

### Study setting

Klivo’s main partners are private health insurance companies in Brazil interested in supporting patients in obtaining consistent results regarding chronic conditions and improving their health-related quality of life. The pilot program has been run in 21 states, but the objective is to reach all the 26 states in this country.

### Study population and design

Patients of all genders and aged ≥ 18 years and <65 years will be enrolled in this study. The International Consortium for Health Outcomes Measurement (ICHOM) will be adopted as a standard for data collection and follow-up [12].

### Patient selection

The private health insurance team members will pre-screen patients diagnosed with T2DM for eligibility. An invitation message will be sent to the identified patients for registration in the KIPDM. The adopted eligibility criteria are as follows.

### Inclusion criteria

1. Diagnosis of T2DM in the electronic medical record of private health insurance companies (HbA1c reading of 7% or higher; age ≥ 18 years and <65 years).
2. Willingness to receive phone calls and messages for monitoring the disease and to check the Klivo app for the educational program.
3. Willingness to use the standard monitoring devices (glucometer), synchronized with the telemonitoring system according to the study protocol throughout the 6-month study period.

### Exclusion criteria

1. Cognitive impairment based on a diagnosis of dementia or mild cognitive impairment reported in the medical records.
2. Self-declared reluctance to receive phone calls or messages for disease management.
3. Pre-existing condition: chronic kidney disease stage 5; individuals with any end-stage disease with a life prognosis of fewer than two years; or pregnant women.

#### Recruitment

The team members of private health insurance companies partnered with the Klivo startup will pre-screen patients diagnosed with T2DM. Patients meeting the eligibility criteria will be invited by text message, during which they will be asked for authorization to share their data for research purposes. Patients will be dismissed from signing a written free informed consent, but the terms of the free informed consent will be available on the first page of the Klivo app.

This protocol was approved by the Research Ethics Committee of Pontifícia Universidade Católica de Minas Gerais (The Pontifical Catholic University of Minas Gerais, Minas Gerais, Brazil) and is registered under number CAAE 53899421.1.0000.5137. It was also approved by the Brazilian Registry of Clinical Trials (registration number RBR-2wdjcdv).

Potential patients who fulfill the eligibility criteria will be provided with answers to their questions related to the consent form. The virtual attending agent will address their questions before the research team obtains their endorsement of informed consent. Also, a contact number will be available for this purpose.

**Fig 1:**
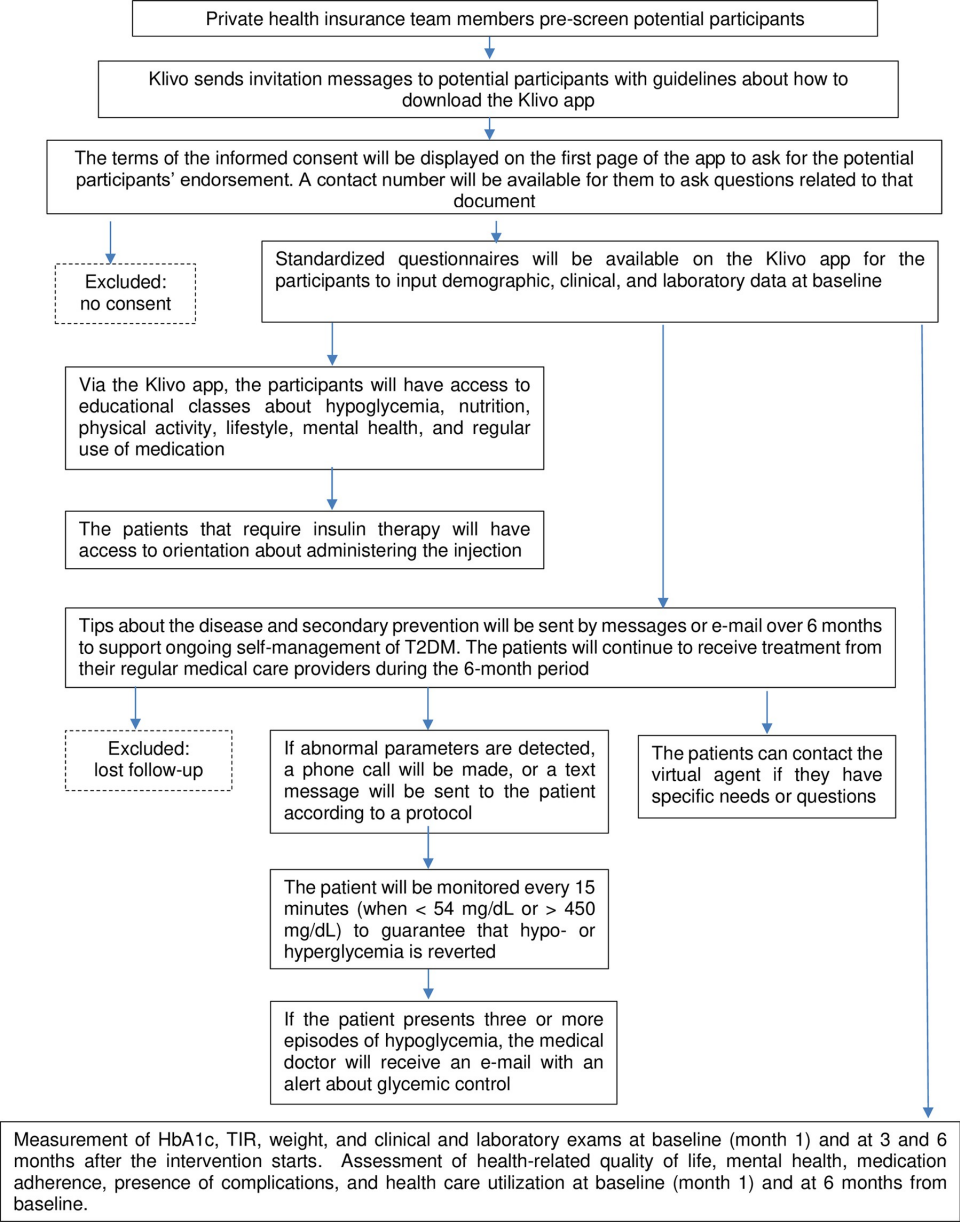
The Klivo Intervention Program (KIPDM) protocol.

All the patients enrolled in the KIPDM will be considered for this cohort, but those who refuse to share their data at any time or lose follow-up will be excluded (**Fig 1**).

### Management process

For 6 months, the patients will be remotely supervised through a management process. This method is an intensive lifestyle intervention program adapted from the National Standards for Diabetes Self-Management Education and Support [13].

The KIPDM will include new technology-based services like smartphone applications and wearable devices, besides traditional interfaces such as phone calls, e-mails, text messages, and the Web (klivo.com). For this cohort, the patients will need to use standardized Bluetooth-enabled devices to transmit their capillary glucose, which will be used for estimating TIR.

### Data collection

HbA1c will be the primary outcome, and some covariates will be evaluated over time (**Table 1**). The secondary outcomes at stipulated time points will include the percentage of TIR. Data will be assessed at baseline (before the intervention) and at 3 and 6 months after the KIPDM starts.

**Table 1:** Data to be evaluated in the timeline of the Klivo Intervention Program (KIPDM).

General information	Diabetes information
**Demographic factors** ^*^	**Diagnosis profile** ^*^
Sex	Diabetes Type
Year of Birth	Year of Diagnosis
Ethnicity/Race	Comorbidities
Education Level	
**Lifestyle and Social Factors** ^*^	**Diabetes Treatment** ^**^
Smoking status	Blood Pressure Lowering Therapy
Baseline and annually	Lipid-lowering Therapy Treatment
Alcohol Consumption	Patient-reported Adherence
Physical Activity	Access to Healthcare
**Patient-reported outcome measures** ^*^	**Diabetes control** ^**^
Psychological well-being (WHO-5)	Glycemic control (HbA1c and TIR)
Diabetes distress (PAID)	Intermediate outcomes (BP, BMI, lipid profile)
Depression (PHQ-9)	Lipodystrophy
	**Chronic complications** ^*^
	Peripheral artery disease
	Ischemic heart disease
	Chronic kidney disease
	Cerebrovascular disease
	Ophthalmopathy

HbA1c, glycated hemoglobin; TIR, time in target blood glucose range; BP, blood pressure; BMI, body mass index; WHO-5, WHO Well-Being Index; PAID, Problem Areas in Diabetes Scale; PHQ-9, Patient Health Questionnaire; ^*^Data will be collected at baseline and at follow-up month 6. ^**^ Data will be collected at baseline and at 3 and 6 months after the intervention starts. Data collection will be based on ICHOM criteria [12].

At 3 and 6 months compared to baseline (month 1), change in weight, percentage of body weight loss, other clinical measurements, and laboratory exams will also be considered. At 6 months compared to baseline, changes in medication adherence, health-related quality of life, mental health status, health care utilization, and clinical complications will be assessed. Other comorbidities such as incident retinal, renal, cardiac, and cerebrovascular complications will also be evaluated. At the end of the KIPDM, the patients will be interviewed so that their experience, acceptance, and perceived usefulness of the KIPDM strategy will be better understood.

### Clinical and laboratory measurements

#### Questionnaires

To obtain information related to the patient’s demographic characteristics, medical history, and environmental risk factors, each patient will fill out the form designed according to the ICHOM criteria [12], on the Klivo app. Additionally, information about physical activity (time per week), smoking status, and alcohol consumption (amount and frequency per week) will be included [12].

To verify medication adherence, data will be collected via a standardized instrument called the Morisky Green scale [14]. The relationship between patients and T2DM will be evaluated by the Problem Areas in Diabetes (PAID) questionnaire [15]. The WHO Well-Being Index (WHO-5) instrument will be used to assess psychological well-being [16]. The depression status will be estimated by the Patient Health Questionnaire (PHQ-9) [17], and strategic directions will be suggested according to the score.

#### Blood pressure measurement

Systolic and diastolic blood pressures will be based on values inserted in the Klivo app by the patient (self-reported).

#### Anthropometric parameters

The patients will report waist circumference (WC) on the Klivo app. To measure WC, the patients will be instructed by a recorded video class. Increased WC will be defined as ≥ 88 cm for women and ≥ 102 cm for men.

The body mass index (BMI) will be calculated as the body weight (kg) divided by the squared height (m^2^). Overweight will be defined as BMI ≥ 25 kg/m^2^ and < 30 kg/m^2^, and obesity will be defined as BMI ≥ 30 kg/m^2^.

#### Biochemical analysis

Fasting blood glucose, HbA1c, total cholesterol, triglycerides, and lipoprotein fractions, as high-density lipoprotein (HDL-c) and low-density lipoprotein (LDL-c), will be measured by standard techniques in the patients’ usual laboratory, every three months.

### Disease diagnosis

Systolic blood pressure (SBP) ≥ 140 mmHg or diastolic blood pressure (DBP) ≥ 90 mmHg (measured in the doctor’s office) or antihypertensive drug use will be used for hypertension diagnosis [18]. T2DM will be defined using American Diabetes Association (ADA) diagnostic criteria, which is in line with the Brazilian Diabetes Society [19, 20]. Dyslipidemia will be defined by drug use (statins and fibrates).

### Statistical analyses plan

By using the R software WebPower package, the sample size was estimated at 1,091. Repeated measure ANOVA with 5 measuring moments, small effect size (0.1), 5% significance level, and power of 80% were considered.

The analysis will be a pre-post design for those members enrolled in the KIPDM. A de-identified dataset of control subjects matched on baseline demographics and clinical characteristics will be cultivated for comparison to the active intervention arm.

For all the measurements, clinical characteristics will be assessed by descriptive statistics. Categorical variables will be expressed as percentages. Continuous variables will be expressed as mean ± SD (standard deviation) or median (interquartile range). The Kolmogorov-Smirnov test will be used to check data normality. The characteristics of the patients in the different groups will be defined according to clinical characteristics. To estimate the p-values, t-test or Wilcoxon rank test (continuous variables) or Pearson’s chi-squared test (categorical variables) will be used. A mixed-effect logistic regression analysis will be used to assess the association between independent variables and T2DM. The analyses will be adjusted for age, sex, and covariates such as hypertension, dyslipidemia, and obesity. Receiver Operational Characteristics (ROC) curves will be performed to evaluate the performance of the models, and the area under the curve (AUC) will be used to measure the discriminatory power of the identified explanatory variables for diabetes. Statistical analysis will be performed by using RStudio software version 1.3.1093. The significance level will be set at 5%.

Sub-groups (e.g., group I and group II) will be created on the basis of pre-existing conditions described as follows.

Group I: Patients without or with only mild non-proliferative diabetic retinopathy without any macular involvement; chronic kidney disease up to stage 3a (eGFR ≥ 45 mL/min/1.73 m^2^); patients without known macrovascular diseases.

Group II: Patients with retinal pathologies documented in the medical records, including proliferative diabetic retinopathy (moderate to severe) or other retinal or macular diseases; or chronic kidney disease stage 3b or 4; or known peripheral vascular, coronary, or cerebrovascular disease.

### Intervention

The intervention for the educational approach will begin with weekly video classes via the Klivo app (**Fig 1**) and will include classes about hypoglycemia, nutrition, physical activity, lifestyle, mental health, and regular use of medication as described below.

#### Video classes

Week 1. Introduction. Orientation about filling out the questionnaires. Orientation about hypoglycemia.
Week 2. Orientation about nutrition
Week 3. Orientation about physical activity.
Week 4. Orientation about medications, especially about the relation between insulin administration time and meals.
Week 5. Orientation about emotional health, smoking, and alcohol.
Week 6. Orientation about healthy changes and glucose control in the long term.

Patients that require insulin therapy will receive one more video class about administering the injection.

#### Monitoring

Other tips about T2DM and secondary prevention will be available on the Klivo app and will be sent to the patients weekly–via e-mail or text message–for six months to support the ongoing self-management of T2DM. All the patients will continue to receive treatment from their regular medical doctors during the 6-month time.

Moreover, depending on the seriousness of the situation, a phone call will be made or a text message will be sent to the patient if abnormal parameters are detected. Phone calls will be made if capillary glucose is < 54 mg/dL or > 450 mg/dL. Text messages will be sent if capillary glucose is between 55 and 70 g/dL or 350 and 449 mg/dL. If capillary glucose is < 54 mg/dL or > 450 mg/dL, the patient will be monitored every 15 minutes to guarantee that hypo- or hyperglycemia is reverted (**Fig 1**).

The patients can also contact the virtual attending agent if they have specific needs or questions.

#### Contacting medical care providers

If the patient presents three or more episodes of hypoglycemia, the medical care provider will receive an e-mail with an alert about glycemic control.

### Pilot sample

While testing the first version of the Klivo app connected to a glucometer sent to the patients’ homes, we retrospectively evaluated data from 107 patients with HbA1c above 7% (mean age 65.86 ±11.10; female 65%; two health insurance companies).

HbA1c and the percentage of TIR readings (measured by capillary glucose) at months 3 or 6 from the baseline were compared to the readings at month 1. HbA1c at baseline (10.54 ± 2.52) differed significantly from HbA1c at month 3 (8.53 ± 1.76; p < 0.001) and 6 (7.42 ± 1.42; p < 0.001).

The percentage of TIR improved significantly going from baseline (60.69 ± 28.67) to month 6 (80.69 ± 24.34; p < 0.001). At month 6 (the last month of evaluation), the patients had blood glucose values within the target range 81% of the time. The main limitation of this analysis was that other factors such as changes in the treatment during the study period were unknown.

A further limitation of the protocol might be the fact that the patient will input anthropometric parameters in the Klivo app, which is not the gold standard.

## Final considerations

In Brazil, KIPDM represents a novelty, and it is the first program for taking care of patients with chronic conditions such as T2DM. This digital service will facilitate data collection related to an individual’s health, helping to evaluate clinical or pre-clinical conditions based on real-world evidence, and will provide personalized intervention and management of the patient’s healthcare.

Establishing a program in which patients are contacted by virtual attending agents and not by bots is challenging. However, we believe that a hybrid model–personal contacts through messages or phone calls and mailing of didactic material and alerts by text messages or e-mail–will enable and enhance patients’ engagement. This program has been designed for T2DM; however, the strategy can be expanded for the challenging management of other chronic conditions.

## Supporting information

S1 Checklist SPIRIT checklist. (PDF)

S1 Protocol Protocol in Portuguese: Protocol approved by the Research Ethics Committee in Portuguese. (PDF)

S2 Protocol Protocol in English: Protocol approved by the Research Ethics Committee translated into English. (PDF)

S1 File Proof of ethics approval in Portuguese: Decision of the Research Ethics Committee in Portuguese. (PDF)

S2 File Proof of ethics approval in English: Decision of the Research Ethics Committee translated into English. (PDF)

S3 File Proof of external funding. (PDF)

S4 File Statement by the study sponsor: Permission from the study sponsor for sharing the protocol publicly under the CC BY 4.0 license. (PDF)
