# Supplementary material for: A data-driven approach to manage type 2 diabetes mellitus through digital health: The Klivo Intervention Program protocol (KIPDM)
Source: PLoS One. 2023 Feb 24;18(2):e0281844. doi: 10.1371/journal.pone.0281844 (PMC9956061; doi:10.1371/journal.pone.0281844)
Supplement: S1 Checklist — (PDF) [file pone.0281844.s001.pdf]

SPIRIT CHECKLIST

[1-5]: ADMINISTRATIVE INFORMATION

1: TITLE

A data-driven approach to manage type 2 diabetes mellitus through digital health: The Klivo Intervention Program protocol (KIPDM)

2: TRIAL REGISTRATION

2A: REGISTRY

ReBEC; Registro Brasileiro de Ensaios Clínicos
(Brazilian Registry of Clinical Trials, at ensaiosclinicos.gov.br)
Registration number: RBR-2wdjcdv

Observation:
ReBEC is listed as a primary registry in the WHO registry network

2B: DATA SET

Data category	Information
Primary registry and trial identifying number	ensaiosclinicos.gov.br<br>RBR-2wdjcdv
Date of registration in primary registry	17 May 2022
Secondary identifying numbers	Open Science Framework<br>10.17605/OSF.IO/5S3K6
Source(s) of monetary or material support	Klivo Licenciamento LTDA.
Primary sponsor	Klivo Licenciamento LTDA.
Secondary sponsor(s)	None
Contact for public queries	André Soares Sá<br>andre.sa@klivo.com<br>+55 11 5507-2497
Contact for scientific queries	Camila Maciel Oliveira, MD<br>camila.maciel@klivo.com<br>+55 11 5507-2497
Public title	The Klivo Intervention Program: a digital platform to monitor patients with type 2 diabetes mellitus
Scientific title	The Klivo Intervention Program protocol: management of type 2 diabetes mellitus through a digital platform
Countries of recruitment	Brazil
Health condition(s) or problem(s) studied	Type 2 diabetes mellitus in adults
Intervention(s)	Digital health program
Key inclusion and exclusion criteria	**Inclusion criteria**<br>Diagnosis of Type 2 diabetes mellitus in the electronic medical record of private health insurance companies (glycated hemoglobin (HbA1c) reading of 7% or higher; age ≥ 18 years and< 65 years)<br><br>Willingness to receive phone calls and messages for monitoring the disease.

	Willingness to use the standard monitoring devices (glucometer), synchronized with the telemonitoring system according to the study protocol throughout the 6-month study period.<br><br>**Exclusion criteria**<br>Cognitive impairment based on a diagnosis of dementia or mild cognitive impairment reported in the medical records<br><br>Self-declared reluctance to receive phone calls or messages for disease management<br><br>Pre-existing condition: chronic kidney disease stage 5; individuals with any end-stage disease with a life prognosis of fewer than two years; or pregnant women
Study type	Interventional (Clinical Trial)<br>Allocation: Single-arm<br>Intervention Model: Parallel Assignment<br>Masking: Single (Investigator)<br>Primary Purpose: Treatment (Digital Health Intervention)
Date of first enrolment	01 November 2022
Target sample size	1,091 patients
Recruitment status	Starting on 01 November 2022
Primary outcome(s)	To evaluate HbA1c values at baseline (before the intervention starts), 3 and 6 months after the start of the intervention. A decrease in HbA1c, measured as percentage, is expected. An 0.4% decrease in HbA1c post-intervention, every three months, will be considered a successful outcome. The goal is a minimum Hb1Ac value of 7%. When this value is reached, the goal will be to maintain this value and not to reduce it even further.
Key secondary outcomes	To evaluate change in time in target blood glucose range (TIR) 3, and 6 months after the intervention starts as compared to baseline. The number of measurements within the ideal blood glucose range is expected to increase. At least a 5% increase in TIR every three months will be considered a successful outcome.
Other outcomes	At 3 and 6 months compared to baseline (month 1): change in weight, percentage of body weight loss, other clinical measurements, and laboratory exams.<br><br>At 6 months compared to baseline (month 1):<br>change in medication adherence, health-related quality of life, mental health status, health care utilization, and clinical complications.

3: PROTOCOL VERSION

Issue Date: 17 May 2022
Protocol Version Number: 01
Author(s): Camila Maciel de Oliveira (MD) and Luiza Borcony Bolognese

4: FUNDING

Klivo Licenciamento Ltda.
CNPJ (Brazilian Corporate Identification Number): 35.996.337/0001-85

5: ROLES AND RESPONSIBILITIES

5A: CONTRIBUTORSHIP

Camila Maciel de Oliveira[1], Luiza Borcony Bolognese[2], Mercedes Bacells[3], Davi Casale Aragon[4], Roberto Luis Zagury[5], Clemente Nobrega[1], Chunyu Liu[6,7]

[1] *Klivo LTDA, São Paulo, Brazil*
[2] *Health Innovation Program, The Pontifical Catholic University of Minas Gerais, Poços de Caldas, Brazil*
[3] *Institute for Medical Engineering and Science, Massachusetts Institute of Technology, Cambridge, USA*
[4] *University of São Paulo, Ribeirão Preto, Brazil*
[5] *Estadual Institut of Diabetes and Endocrinology Luiz Capriglione (IEDE), Rio de Janeiro, Brazil*
[6] *Framingham Heart Study, Framingham, USA*
[7] *Department of Biostatistics, Boston University, Boston, USA*

Conceptualization: CMO and CN
Data curation: CMO and DCA
Investigation: CMO, LBB, MB, and CL
Methodology: CMO, DCA, and CN
Supervision: CMO
Validation: CMO, CN, DCA, and RLZ
Writing – original draft: CMO, LBB, and CN
Writing – review & editing: CMO, MB and CL

5B: SPONSOR CONTACT INFORMATION

Klivo Licenciamento LTDA
Contact name:
Mr. André Soares Sá
Address:
Rua Afonso Braz, 373
Vila Nova Conceição
São Paulo - SP,
Brasil
CEP 04511-011
Telephone:
+55 11 5507-2497
Email:
andre.sa@klivo.com

5C: SPONSOR AND FUNDER

This funding source had no role in the design of this study and will not have any role during its analyses, data interpretation, or decision to submit results.

5D: COMMITTEES
Not applicable.

[6-8]: INTRODUCTION

6: INTRODUCTION

6A: BACKGROUND AND RATIONALE
Digital therapeutics – an emerging type of medical approach that is expanding globally with market demand – is defined as evidence-based therapeutic interventions through qualified software programs that help prevent, manage, and treat chronic diseases [1]. Some studies have suggested that continuous remote evaluation and daily monitoring can effectively refine the management of chronic conditions such as type 2 diabetes mellitus (T2DM), which has a high prevalence and social and economic burden [2,3]. In this sense, digital therapeutics products addressed to individual needs have contributed to ongoing management of daily life habits and to lowering healthcare costs attributed to chronic metabolic diseases [4, 5].

These technologies have improved awareness about diet and regular exercise, optimized glycemic control, and ensured adherence to medication use, consequently lowering the high cost of treating T2DM [6]. Indeed, clinical advancement of patients in protocols such as the Livongo for Diabetes Program has lowered average costs per patient per month by $83 [7], which is crucial if we consider the estimation that 700 million people will be living with diabetes in 2045 [2]. Therefore, programs that support lifestyle changes can perform secondary prevention by reducing the risk of chronic complications or even primary prevention by delaying disease diagnosis [8].

There is a consensus that value is created by enabling health and not by just delivering care. Some healthcare organizations have offered digital programs as part of the value-based care model [9]. Furthermore, professionals who use digital health technologies benefit from short-term and long-term health improvements [10]. Therefore, here we intend to describe the use of a digital health strategy – the Klivo Intervention Program for T2DM (KIPDM). This study seeks to assess an intensive lifestyle intervention method that offers virtual support for patients with T2DM through continuous remote evaluation and weekly monitoring, which will contribute to managing T2DM by improving essential issues related to this disease. Additionally, the support for lifestyle changes can reduce the risk of other related chronic complications. A better understanding of the efficacy of a digital health program will directly benefit the patients of this study and contribute to the design of scalable solutions for chronic disease.

6B: CHOICE OF COMPARATORS
No comparator groups will be used in this study [11-13].

7: OBJECTIVES
Research Hypothesis
KIPDM patients should present improved HbA1c and TIR along the intervention as compared to baseline values.

Main objective:
To evaluate aspects related to glycemic control of patients with T2DM recruited for the KIPDM cohort. Additionally, self-reported clinical outcomes, health-related quality of life, mental health, medication adherence, and healthcare utilization will be evaluated

by using validated questionnaires. Glycated hemoglobin (HbA1c) will be evaluated as the primary outcome. The time in range (TIR), defined as the percentage of time an individual remains with blood glucose levels in a target range of 70 to 180 mg/dL, will be evaluated as a secondary outcome.

Specific objectives:

1. To compare the patient's HbA1c values at baseline, 3, and 6 months after inclusion in KIPDM.
2. To identify the percentage of time in range (TIR) and, hence, the number of hypoglycemic events (< 70mg/dL) over 6 months.

Secondary objectives:

To evaluate change in weight, body weight loss, and other clinical measurements and laboratory exams at 3 and 6 months compared to baseline.

To evaluate changes in health-related quality of life, mental health status, medication adherence, health care utilization, and clinical complications at 6 months compared to baseline.

8: TRIAL DESIGN

Study Design

Study Type: Interventional (Clinical Trial)
Estimated Enrollment: 1,091 patients
Allocation: Single-arm
Intervention Model: Parallel Assignment
Masking: Single (Investigator)
Primary Purpose: Treatment (Digital health intervention)
Official Title: Klivo Intervention Program
Actual Study Start Date: 01 November 2022
Estimated Primary Completion Date: July 2023
Estimated Study Completion Date: July 2024

Arms and Interventions

Arms

Experimental: Klivo Intervention Program for Type 2 diabetes mellitus (KIPDM)
The program will be based on a 6-month management process during which patients will receive video classes for health education, online access to a live virtual agent, and a device for monitoring glucose levels.

Behavioral: Klivo Intervention Program (KIPDM)
The patients will receive a glucometer for monitoring glucose levels that will be connected to the Klivo app. When abnormal parameters are detected, patients and medical care providers will be contacted according to an established protocol. Clinical and laboratory data, weight control, health-related quality of life, mental health, medication adherence, and healthcare utilization will be evaluated through validated electronic questionnaires at baseline and six months after inclusion in KIPDM. The program will include educational videos via messages through the app to approach specific issues, over the six months. Then, tips about the disease and secondary prevention will be sent to patients also through the app.

[9-15]: METHODS: PATIENTS, INTERVENTIONS, OUTCOMES

9: STUDY SETTING

Klivo is a Brazilian startup founded in 2020 and certified by the Brazilian Society of Diabetes in February 2022. KIPDM seeks to follow up patients with chronic conditions in their self-care along time. Its main partners are private health insurance companies in Brazil. The Klivo team supports individuals in obtaining consistent results in metabolic conditions and improving their health-related quality of life. This digital platform will facilitate data collection related to an individual's health, analyze data to evaluate clinical or pre-clinical conditions, and provide personalized management of the patient's treatment for 6 months.

10: ELIGIBILITY CRITERIA

Inclusion criteria

1. Diagnosis of T2DM in the electronic medical record of private health insurance companies (HbA1c reading 7% or higher; age ≥ 18 years and <65 years).
2. Willingness to receive phone calls and messages for monitoring the disease and education classes.
3. Willingness to use the standard monitoring devices (glucometer), synchronized with the telemonitoring system according to the study protocol throughout the 6-month study period.

Exclusion criteria

1. Cognitive impairment based on a diagnosis of dementia or mild cognitive impairment reported in the medical records.
2. Self-declared reluctance to receive phone calls or messages for disease management.
3. Pre-existing condition: chronic kidney disease stage 5; patients with any end-stage disease with a life prognosis of fewer than two years; or pregnant women.

11: INTERVENTION (INTERVENTIONS, MODIFICATIONS, ADHERENCE, CONCOMITANT CARE)

The intervention for the educational approach will begin with weekly video classes via the KIP app.

It will include recorded classes about hypoglycemia, nutrition, physical activity, lifestyle, mental health, and regular use of medication as described below.

Classes

1. Introduction. Orientation for filling out the questionnaires. Orientation about hypoglycemia.
2. Orientation about nutrition
3. Orientation about physical activity.
4. Orientation about medications, especially about the relationship between insulin administration time and meals.
5. Orientation about emotional health, smoking, and alcohol.
6. Orientation about healthy changes and glucose control in the long term.

Patients that require insulin therapy will receive other video class about administering the injection.

Monitoring

Other tips about T2DM and secondary prevention will be available on the App and will be sent to the patients weekly for six months to support ongoing self-management of T2DM. All the patients will continue to receive treatment from their regular medical doctors during the 6-month time.

Moreover, depending on the seriousness of the situation, a phone call will be made or a text message will be sent to the patients if abnormal parameters are detected. Phone calls will be made if capillary glucose is < 54 mg/dL or > 450 mg/dL, and text messages will be sent if capillary glucose is between 55 and 70 g/dL or 350 and 449 mg/dL. If capillary glucose is < 54 mg/dL or > 450 mg/dL, the patients will be monitored every 15 minutes to guarantee that hypo- or hyperglycemia is reverted.

The patients can contact the virtual agent if they have specific needs or questions.

Contacting medical doctors
If a patient presents three or more episodes of hypoglycemia, their medical doctor will receive an e-mail with an alert about their glycemic control.

Clinical and laboratory measurements
Questionnaires
To obtain information related to the patients' demographic characteristics, medical history, and environmental risk factors, patients will answer a questionary designed according to the ICHOM criteria by inputting the information on Klivo app [14]. Additionally, information about physical activity (times per week), smoking status, and alcohol consumption (amount and frequency per week) will be also answered according to these criteria [14].

To verify medication adherence, data will be collected via a standardized instrument called the Morisky Green scale [15]. The relationship between patients and T2DM will be evaluated by the Problem Areas in Diabetes (PAID) questionnaire [16]. The WHO Well-Being Index (WHO-5) instrument will be used to assess psychological well-being [17]. The depression status will be estimated by the Patient Health Questionnaire (PHQ-9) [18], and strategic directions will be suggested according to the score.

Blood pressure measurement and anthropometric parameters

Systolic and diastolic blood pressures will be based on values entered by the patient in the Klivo app (self-reported). Patients will be asked by the app to report all the anthropometric parameters. To measure waist circumference (WC), patients will be instructed by a video class.

Biochemical analysis
Blood glucose, HbA1c, total cholesterol, triglycerides, and lipoprotein fractions (as high-density lipoprotein, HDL-c; and low-density lipoprotein, LDL-c) will be measured by standard techniques in the patient's usual laboratory, every three months, and will be registered during the phone call or through the Klivo app.

Disease diagnosis
Increased WC will be defined as ≥ 88 cm for women and ≥ 102 cm for men [19].

Body mass index (BMI) will be calculated as the body weight (kg) divided by the squared height (m^2). Overweight will be defined as BMI ≥ 25 kg/m^2 and < 30 kg/m^2, and obesity will be defined as BMI ≥ 30 kg/m^2.

Systolic blood pressure (SBP) ≥ 140 mmHg or diastolic blood pressure (DBP) ≥ 90 mmHg (measured in the doctor's office or at home) or antihypertensive drug use will define the hypertension diagnosis [20]. T2DM will be defined using the American Diabetes Association (ADA) diagnostic criteria, which is in line with the Brazilian Diabetes Society. Dyslipidemia will be defined by drug use (statins or fibrates).

12: OUTCOMES

Expected academic results:
KIPDM patients should present improved HbA1c and TIR along the intervention as compared to baseline values.

Primary Outcome:
To evaluate change in glycated hemoglobin (HbA1c) values comparing baseline (before the intervention starts), 3 and 6 months after the start of the intervention will be analyzed. A decrease in HbA1c, measured as a percentage, is expected. HbA1c will be evaluated by laboratory exams requested by the patient's medical doctor. The Hb1Ac values will be obtained via Klivo app at 3 and 6 months after the intervention starts and will be compared to the Hb1Ac at baseline. A 0.4% decrease in HbA1c post-intervention, every three months, will be considered a successful outcome. The goal is a minimum Hb1Ac value of 7%. When this value is reached, the goal will be to maintain this value and not to reduce it even further [21].

Secondary outcome:
To evaluate change in time in target blood glucose range (TIR), values on 3 and 6 months after the intervention starts will be compared to baseline. The number of measurements within the ideal blood glucose range is expected to increase. To evaluate this outcome, capillary glucose, in mg/dL, will be measured with a glucometer, which will be sent to the patient's home address. The glucose level will be obtained with an app downloaded on the patient's smartphone, connected to the glucometer. At least a 5% increase in TIR every three months will be considered a successful outcome.

Other Outcomes:
Change in weight at 3 and 6 months compared to baseline (month 1).
Change in percentage of body weight loss at 3 and 6 months compared to baseline (month 1).
Change in other clinical measurements (blood pressure, waist circumference) and laboratory exams (total cholesterol, HDL-c, LDL-c, triglycerides, creatinine).
The presence of retinal, renal, cardiac, and cerebrovascular complications at baseline and month 6.
Change in medication adherence at month 6 compared to baseline (month 1).
Change in health-related quality of life at month 6 compared to baseline (month 1).
Change in mental health status at month 6 compared to baseline (month 1).
Health care utilization at month 6 compared to baseline (month 1).

13: PATIENT TIMELINE

Private health insurance team members pre-screen potential participants

Klivo send invitation messages to potential participants for orienting how to download the app

The terms of the informed consent will be on the first page of the app to ask for the potential participants' endorsement. A contact number will be available to answer all their questions related to that document

Excluded: no consent

Standardized questionnaires will be available on the Klivo app for recording demographic, clinical and laboratory data at baseline

Patients will have access to educational video classes weekly over six months via Klivo app about hypoglycemia, nutrition, physical activity, lifestyle, mental health, and regular use of medication

Patients that require insulin therapy take other orientation classes about administering the injection

Tips about T2DM and secondary prevention will be sent by messages or e-mail weekly over six months to support ongoing self-management of T2DM. Patients continue to receive treatment from their regular medical care providers during the 6-month time

Excluded: lost follow-up

If abnormal parameters are detected, a phone call will be made, or a text message will be sent to the patient

The patients can contact the virtual agent if they have specific needs or questions.

Patients will be monitored every 15 minutes (when < 54 mg/dL or > 450 mg/dL) to guarantee that hypo- or hyperglycemia is reverted

If the patient presents three or more episodes of hypoglycemia (<54mg/dL), the medical doctor will receive an e-mail with an alert about glycemic

Measurement of HbA1c, TIR, weight, clinical and laboratory exams at baseline (month 1), 3, and 6 months. Assessment of health-related quality of life, mental health, medication adherence, presence of complications, health care utilization, and at baseline (month 1) and 6 months from baseline.

14: SAMPLE SIZE
By using the R software WebPower package, the sample size has been estimated at 1,091. The following was considered: repeated measure ANOVA with 5 measuring moments, small effect size (0.1), 5% significance level, and power of 80%.

15: RECRUITMENT
The team members of private health insurance companies partnered with the Klivo startup will pre-screen patients diagnosed with T2DM. After this stage, the eligibility criteria will be used. Patients meeting the eligibility criteria will be invited by text message, during which they will be asked for authorization to share their data for statistical analysis and results publications. Patients will be dismissed from signing a written free informed consent, but the terms of the free informed consent will be available on the first page of the Klivo app.

[16-17]: METHODS: ASSIGNMENTS OF INTERVENTIONS (FOR CONTROLLED TRIALS)
Not Applicable

[18-20]: METHODS: DATA COLLECTION, MANAGEMENT, ANALYSIS

18: DATA COLLECTION METHODS

The intervention for the educational approach will begin with weekly video classes via the KIP app.

It will include classes about hypoglycemia, nutrition, physical activity, lifestyle, mental health, and regular use of medication as described below.

Classes
1. Introduction. Questionnaires. Orientation about hypoglycemia.
2. Orientation about nutrition
3. Orientation about physical activity.
4. Orientation about medications, especially about the relationship between insulin administration time and meals.
5. Orientation about emotional health, smoking, and alcohol.
6. Orientation about healthy changes and glucose control in the long term.

Patients that require insulin therapy will receive one more video class about administering the injection.

Monitoring
Other tips about diabetes mellitus and secondary prevention will be available on the App and will be sent to the patients weekly for six months to support ongoing self-management of T2DM. All the patients will continue to receive treatment from their regular medical doctors during the 6-month time.

Moreover, depending on the seriousness of the situation, a phone call will be made or a text message will be sent to the patients if abnormal parameters are detected. Phone calls will be made if capillary glucose is < 54 mg/dL or > 450 mg/dL, and text messages will be sent if capillary glucose is between 55 and 70 g/dL or 350 and 449 mg/dL. The patients will be monitored every 15 minutes to guarantee that hypo- or hyperglycemia is reverted, when capillary glucose is < 54 mg/dL or > 450 mg/dL.

The patients can contact the nurse team if they have specific needs or questions.

Contacting medical doctors

If a patient presents three or more episodes of hypoglycemia (< 54 mg/dL), their medical doctor will receive an e-mail with an alert about their glycemic control.

Clinical and laboratory measurements

Questionnaires

To obtain information related to the patients' demographic characteristics, medical history, and environmental risk factors, patients will answer a questionary designed according to the ICHOM criteria [14] by inputting the information on Klivo app. Additionally, information about physical activity (times per week), smoking status, and alcohol consumption (amount and frequency per week) will be also answered according to these criteria [14].

To verify medication adherence, data will be collected via a standardized instrument called the Morisky Green scale [15]. The relationship between patients and T2DM will be evaluated by the Problem Areas in Diabetes (PAID) questionnaire [16]. The WHO Well-Being Index (WHO-5) instrument will be used to assess psychological well-being [17]. The depression status will be estimated by the Patient Health Questionnaire (PHQ-9) [18], and strategic directions will be suggested according to the score.

Blood pressure measurement and anthropometric parameters

Systolic and diastolic blood pressures will be based on values entered by the patient in the Klivo app (self-reported). Patients will be asked by the app to report all the anthropometric parameters. To measure waist circumference (WC), patients will be instructed by a video class.

Biochemical analysis

Blood glucose, HbA1c, total cholesterol, triglycerides, and lipoprotein fractions (as high-density lipoprotein, HDL-c; and low-density lipoprotein, LDL-c) will be measured by standard techniques in the patient's usual laboratory exams, every three months, and will be registered by the patient through the Klivo app.

Disease diagnosis

Increased WC will be defined as ≥ 88 cm for women and ≥ 102 cm for men [19].

Body mass index (BMI) will be calculated as the body weight (kg) divided by the squared height (m^2). Overweight will be defined as BMI ≥ 25 kg/m^2 and < 30 kg/m^2, and obesity will be defined as BMI ≥ 30 kg/m^2.

Systolic blood pressure (SBP) ≥ 140 mmHg or diastolic blood pressure (DBP) ≥ 90 mmHg (measured in the doctor's office or at home) or antihypertensive drug use will define the hypertension diagnosis [20]. T2DM will be defined using American Diabetes Association (ADA) diagnostic criteria, which is in line with the Brazilian Diabetes Society [19, 20]. Dyslipidemia will be defined by drug use.

19: DATA MANAGEMENT

The information obtained in this study will be confidential. Confidentiality about participation will be ensured in all stages of the research and during the presentation

of the results in scientific or educational journals given that the results will always be presented as representative of a group and not a person.

All the material collected during the research will be kept by and will remain under the responsibility of the Klivo Research Center for two years. After that, the material will be filed for an indeterminate time and will remain under the responsibility of Klivo.

20: STATISTICAL METHODS

By using the R software WebPower package, the sample size has been estimated at 1,091. The following was considered: repeated measure ANOVA with 5 measuring moments, small effect size (0.1), 5% significance level, and power of 80%.

The analysis will be a pre-post design for those members enrolled in KIPDM. A de-identified dataset of control subjects matched on baseline demographics and clinical characteristics will be cultivated for comparison to the active intervention arm.

For all the measurements, clinical characteristics will be assessed by descriptive statistics. Categorical variables will be expressed as percentages, and continuous variables will be expressed as mean ± SD or median (interquartile range). The Kolmogorov-Smirnov test will be used to check data normality. The characteristics of the patients in the different groups (defined according to clinical characteristics) will be evaluated by t-test or Wilcoxon rank test (continuous variables) or Pearson's chi-squared test (categorical variables). A mixed-effect logistic regression analysis will be used to assess the association between independent variables and T2DM. The analyses will be adjusted for age, sex, and covariates such as hypertension, dyslipidemia, and obesity. Receiver Operational Characteristics (ROC) curves will be performed to evaluate the performance of the models, and the area under the curve (AUC) will be used to measure the discriminatory power of the identified explanatory variables for diabetes. Statistical analysis will be performed by using RStudio software version 1.3.1093. The significance level will be set at 5%.

Sub-groups (e.g., group I and group II) will be created on the basis of pre-existing conditions described as follows.

Group I: Patients with no or only mild non-proliferative diabetic retinopathy without any macular involvement; chronic kidney disease up to stage 3a (eGFR ≥ 45 mL/min/1.73 m2); patients with no known macrovascular diseases.

Group II: Patients with retinal pathologies documented in the medical records, including proliferative diabetic retinopathy (moderate to severe) or other retinal or macular diseases; or chronic kidney disease stage 3b or 4; or known peripheral vascular, coronary, or cerebrovascular disease.

Primary Outcome:
To evaluate glycated hemoglobin (HbA1c) values at baseline (before the intervention starts), 3 and 6 months after the start of the intervention. A decrease in HbA1c, measured as a percentage, is expected. HbA1c will be evaluated by laboratory exams requested by the patient's medical doctor. The Hb1Ac values will be obtained via a questionnaire answered by the patient during a phone call or via de Klivo app at 3 and 6 months after the intervention starts and will be compared to the Hb1Ac at baseline. A 0.4% decrease in HbA1c post-intervention, every three months, will be considered a successful outcome. The goal is a minimum Hb1Ac value of 7%. When this value is reached, the goal will be to maintain this value and not to reduce it even further [21].

Secondary outcome:
To evaluate change in time in target blood glucose range (TIR), values on 3 and 6 months after the intervention starts will be compared to baseline. The number of measurements within the ideal blood glucose range is expected to increase. To

evaluate this outcome, capillary glucose, in mg/mL, will be measured with a glucometer, which will be sent to the patient's home address. The glucose level will be obtained with an app downloaded on the patient's smartphone, connected to the glucometer. At least a 5% increase in TIR every three months will be considered a successful outcome.

Other Outcomes:
Change in weight at 3 and 6 months compared to baseline (month 1).
Change in percentage of body weight loss at 3 and 6 months compared to baseline (month 1).
Change in clinical measurements (blood pressure, waist circumference) and laboratory exams (total cholesterol, HDL-c, LDL-c, triglycerides, creatinine).
The presence of retinal, renal, cardiac, and cerebrovascular complications at baseline and month 6.
Change in medication adherence at month 6 compared to baseline (month 1).
Change in health-related quality of life at month 6 compared to baseline (month 1).
Change in mental health status at month 6 compared to baseline (month 1).
Health care utilization at month 6 compared to baseline (month 1).

[21-23] METHODS: MONITORING

21: DATA MONITORING

21A: FORMAL COMMITTEE

An audit service will evaluate the data process and analysis for improving the effectiveness of risk management.

21B: INTERIM ANALYSIS
Not applicable

22: HARMS
The program involves educational actions in health and monitoring (especially glycemic control). Risks are related to disease diagnosis (for example, hypo- or hyperglicemia) and measures to mitigate such events. There are also risks involved with data handling (leak, hacker attack, risks to safety and privacy, for instance).

23: AUDITING
An audit service will evaluate the data process and analysis for improving the effectiveness of risk management.

[24-31]: ETHICS AND DISSEMINATION

24: RESEARCH ETHICS APPROVAL
This protocol was approved by the Research Ethics Committee of Pontifícia Universidade Católica de Minas Gerais (The Pontifical Catholic University of Minas Gerais, Minas Gerais, Brazil) and is registered under number 5.246.322

25: PROTOCOL AMENDMENTS

Any modifications to the protocol that may impact the conduct of the study, potential patient's benefit, or patient's safety, including changes in study objectives, study design, patient population, sample sizes, study procedures, or significant administrative aspects will require a formal amendment to the protocol. Such amendment will be agreed upon by the Ethics Committee prior to implementation and notified to the health authorities in accordance with local regulations.

26: CONSENT OR ASSENT

26A: CONSENT OR ASSENT:

The team members of private health insurance companies partnered with the Klivo startup will pre-screen patients diagnosed with T2DM. After this stage, the eligibility criteria will be used. Patients meeting the eligibility criteria will be invited by text message, during which they will be asked for authorization to share their data for statistical analysis and results publications. Patients will be dismissed from signing a written free informed consent, but the terms of the free informed consent will be available on the Klivo app.

26B: ANCILLARY STUDIES

If an ancillary study derives from the present trail, a new consent will be obtained from every patient in the ancillary study, if the data collection/request is not covered in the original informed consent process.

27: CONFIDENTIALITY

The authors of the research will only analyze the data made available by the partner startup (Klivo), which monitors patients with diabetes mellitus through a digital platform. The protocol developed and operated by the research team will only be used for statistical analyses and scientific publication. The authors will receive encrypted data, while the patients' identity will remain totally anonymous. In other words, the authors of the research assure the Research Ethics Committee that the patients' identity will remain anonymous and protected. If any clinical records or other documents are submitted by the partner, they will be identified by a code and not by the patient's name.

28: DECLARATION OF INTEREST

Four authors were consultants or collaborators to Klivo, the funder of this study.
Three authors are independent researchers.

29: ACCESS TO DATA

Any data reported in this study will be fully available.

30: ANCILLARY AND POST-TRIAL CARE

Not applicable.

31: DISSEMINATION POLICY

31A. TRIAL RESULTS

Results will be presented at international and national conferences, symposia, congresses, and meetings. Results will be registered on the ReBEC (Brazilian Registry of Clinical Trials). Results will be published in Scientific Journals.

31B. AUTHORSHIP

Substantive contributions to the design, conduct, interpretation, and reporting of the results of this research will be recognized by granting the authorship in a way that

ensures transparency and avoids disputes or misunderstandings after research completion.

Individuals who fulfill authorship criteria will not remain hidden (ghost authorship) and will have final authority over manuscript content. Individuals who do not fulfill such criteria will not be granted authorship (guest authorship).

31C. REPRODUCIBLE RESEARCH

Not applicable

[32]: APPENDICES

32: INFORMED CONSENT MATERIALS

Below you will find the Free Informed Consent Term.

FREE INFORMED CONSENT TERM

Plataforma Brasil Registration Number: CAAE 53899421.1.0000.5137
Research Ethics Committee Registration Number: 5.246.322

Title of Project: **The Klivo Intervention Program protocol: management of type 2 diabetes mellitus through a digital platform**

Dear Sir/Madam,

You are being invited to participate in a research project that will study a program to monitor people's health. The program will help to monitor chronic diseases, like diabetes. The program will use technology that has helped improve awareness of the importance of healthy eating habits, keeping physically active, and controlling glucose levels, which also helps reduce the risk of complications of other diseases.

You have been selected because your medical chart contains diagnosis of diabetes with glycated hemoglobin of 7% or higher and you are aged 18 years or older. In addition, you have been selected because you are willing to receive phone calls and messages for monitoring the disease and for tele-education. Another reason why you have been selected is that you are willing to use the glucometer to monitor glucose, synchronized with the telemonitoring system, throughout the 12-month study period.

Klivo's webpage is www.klivo.com and it will always be available for you to obtain additional information and send questions. More specifically, your participation in this study consists in answering questionnaires, via a phone call, for us to record your demographic data (such as date of birth and address), clinical information (such as weight and height), information about your lifestyle (such as eating habits, physical activity, smoking, and alcohol consumption), and transmission of laboratory exam results (such as glycated hemoglobin and cholesterol). Other information will also be collected. You'll answer questions about your quality of life, use of medications, and emotional aspects that may interfere in the follow-up of your disease.

In the first 6 weeks, nurses working at Klivo will contact you via a telephone call or messages once a week to conduct the so-called health education or tele-education. You will receive information about the following topics: how to prevent hypoglycemia; nutrition and physical activity; use of medications; emotional health, smoking, and alcohol; and importance of keeping healthy habits and controlling glucose levels in the long term. If you use insulin, you will be offered an additional session about insulin administration. Along 12 months, you will be contacted at least every 15 days so that we can ask you about occasional complaints or news about your treatment.

You will also be asked to enable your cell phone Bluetooth for transmission of capillary glucose. If your glucose level is too high or too low, a Klivo team member will get in touch with you within 3 minutes by phone or text message. If your glucose level is lower than 54 or higher than 450, you will receive a phone call. If your glucose level is between 55 and 70 or between 350 and 449, you will receive a text message. The Klivo team will contact you again after 15 minutes to ensure that your glucose level has normalized or to suggest other measures. If specific needs are detected, the Klivo team will suggest scheduling additional calls with the multidisciplinary team, which includes psychologists, nutritionists, and physical educators.

If you present three or more hypoglycemic (low glucose levels) or hyperglycemic (high glucose levels) events, your medical doctor will receive an e-mail message with a report about your glycemic control.

All the information collected about you will be encrypted and kept confidential for exclusive use of the Klivo Research Center. When your information is distributed to other team members or parties for statistical analysis, all your data will be anonymous to preserve your privacy.

There are two main risks (and/or discomfort) involved in this study. The first is the chance that the data storage system be invaded, and your information be seen by other people. The second is the risk of hypoglycemia – or low glucose levels – and this could happen if you ate less food than recommended, skipped meals, allowed a longer time between meals than recommended, did not eat a snack between meals, or started more intense physical activity than you are used to.

Your participation is very important and voluntary, so you will not be paid for participating in the study. On the other hand, you will have no expenses.

The information obtained in this study will be confidential. Confidentiality about your participation will be ensured in all stages of the research and during presentation of the results in scientific or educational journals, given that the results will always be presented as representative of a group and not a person. You may refuse to participate or to answer questions at any time, without any personal losses.

All the material collected during the research will be kept by and will remain under the responsibility of the main investigator for 2 years. After that, the material will be filed for indeterminate time and will remain under the responsibility of Klivo.

The results of this research will directly benefit the patients of the research, who will be closely monitored and will follow a protocol that is close to the ideal protocol advocated for patients with diabetes. The results of this research will also benefit other patients with diabetes because they will provide relevant information of the best way for delivering care to these patients.

Under the law, all the patients are covered by civil liability in case of damages due to the research.

A copy of this term containing the contact information for the main investigator will be sent to your home address and you should keep it. You can ask any questions about the project and your participation in it at any time.

Main investigator: Camila Maciel de Oliveira, email address: camila.maciel@klivo.com.

This study was approved by the Research Ethics Committee of Pontifícia Universidade Católica de Minas Gerais (The Pontifical Catholic University of Minas Gerais), coordinated by Prof. Cristiana Leite Carvalho, who, in case of ethical issues, can be contacted at (31)3319-4517 or cep.proppg@pucminas.br.

The Research Ethics Committee is a local authority and gateway for research projects involving humans. The aim of the Committee is to stand up for the rights and interest of the patients, ensuring their integrity and dignity and contributing to the development of research within ethical standards.

You can now tell us if you agree to participate in the research on the basis of the information recorded in this free informed consent term. In case you do not agree to participate, tell us

about your decision at this time, so that we will maintain your follow-up without your being included in the research.

I, **Camila Maciel de Oliveira**, am committed to complying with all the requirements and duties described in this term and I am grateful for your collaboration and trust.

	31 May 2022
Investigator's signature	Date

REFERENCES

[1] Hong JS, Wasden C, Han DH. Introdução da terapêutica digital. Programas de Métodos computacionais Biomed. 2021; 209: 106319. pmid: 34364181.

[2] Saeedi P, Petersohn I, Salpea P, Malanda B, Karuranga S, Unwin N, et al; Comitê atlas de diabetes do IDF. Estimativas de prevalência global e regional de diabetes para 2019 e projeções para 2030 e 2045: Resultados do Atlas da Federação Internacional de Diabetes, 9ª edição. Diabetes Res Clin Pract. 2019; 157: 107843. pmid: 31518657.

[3] Rhee SY, Kim C, Shin DW, Steinhubl SR. Presente e futuro da saúde digital em diabetes e doença metabólica. Diabetes Metab J. 2020; 44(6): 819-27. pmid: 33389956.

[4] Thorpe K, Toles A, Shah B, Schneider J, Bravata MD. Redução associada à perda de peso nos gastos com assistência médica para indivíduos com seguro comercial com condições crônicas. J Occup Environ Med. 2021; 16 de junho. doi: 10.1097/jom.0000000000002296. Epub à frente da impressão. pmid: 34138824.

[5] Huckfeld PJ, Frenier C, Pajewski NM, Espeland M, Peters A, Casanova R, et al. Associações de intervenção intensiva do estilo de vida no diabetes tipo 2 com uso de cuidados de saúde, gastos e incapacidade: estudo auxiliar do estudo look AHEAD. JAMA Netw Open. 2020; 3(11): e2025488. pmid: 33231638.

[6] Downing J, Bollyky J, Schneider J. Uso de um medidor de glicose conectado e treinamento certificado de educador de diabetes para diminuir a probabilidade de excursões anormais de glicose: o Programa Livongo para Diabetes. J Med Internet Res. 2017; 19(7): e234. pmid: 28698167.

[7] Bollyky JB, Bravata D, Yang J, Williamson M, Schneider J. Coaching de estilo de vida remoto, além de um medidor de glicose conectado com suporte certificado de educador de diabetes melhora a glicose e a perda de peso para pessoas com diabetes tipo 2. J Diabetes Res. 2018: 3961730. pmid: 29888288.

[8] Goh KLS, Lee CS, Koh CHG, Ling NL, Ang SB, Oh C. Avaliando a eficácia e a utilidade de um novo sistema de telemonitoramento culturalmente adaptado para melhorar o controle glicêmico dos asiáticos com diabetes mellitus tipo 2: um protocolo de estudo de método misto. Ensaios. 2021; 22(1): 305. pmid: 33902656.

[9] Porter ME, Teisberg EO. Redefinição da saúde - criando concorrência baseada em valor em resultados. Boston: Harvard Business School Press; 2006.

[10] Kaufman N, Khurana I. Utilizando tecnologia digital de saúde para prevenir e tratar o diabetes. Diabetes Technol Ther. 2016; 18 Suppl 1 (Suppl 1): S56-S68. pmid: 26836430.

[11] Zheng L, Rosenkranz SL, Taiwo B, Para MF, Eron JJ Jr, Hughes MD. The design of single-arm clinical trials of combination antiretroviral regimens for treatment-naive HIV-infected patients. AIDS Res Hum Retroviruses. 2013; 29(4):652-7. pmid: 23228206.

[13] Arima M, Inoue H, Nakao S, Misumi A, Suzuki M, Matsushita I, Araki S, Yamashiro C, Takahashi K, Ochiai M, Yoshida N, Hirose M, Kishimoto J, Todaka K, Hasegawa S, Kimura K, Kusuhara K, Kondo H, Ohga S, Sonoda KH. Study protocol for a multicentre, open-label, single-arm phase I/II trial to evaluate the safety and efficacy of ripasudil 0.4% eye drops for retinopathy of prematurity. BMJ Open. 2021; 11(7):e047003. pmid: 34315793.

[14] Nano J, Carinci F, Okunade O, Whittaker S, Walbaum M, Barnard-Kelly, K, et al.; Grupo de Trabalho de Diabetes do Consórcio Internacional de Medição de Resultados em Saúde (ICHOM). Um conjunto padrão de desfechos centrados nas pessoas para diabetes mellitus: resultados de uma abordagem internacional e unificada. Diabet Med. 2020; 37(12): 2009-18. pmid: 3214488.

[15] Morisky DE, Green LW, Levine DM. Concurrent and predictive validity of a self-reported measure of medication adherence. Med Care. 1986; 24(1): 67-74. pmid: 3945130.

[16] Schmitt A, Reimer A, Kulzer B, Haak T, Ehrmann D, Hermanns N. How to assess diabetes distress: comparison of the Problem Areas in Diabetes Scale (PAID) and the Diabetes Distress Scale (DDS). Diabet Med. 2016; 33(6): 835-43. pmid:26287511.

[17] Topp CW, Østergaard SD, Søndergaard S, Bech P. The WHO-5 Well-Being Index: a systematic review of the literature. Psychother Psychosom. 2015; 84(3): 167-76. pmid: 25831962.

[18] Koenke K, Spitzer RL, Williams JBW. The PHQ-9 validity of a brief depression severity measure. J Gen Intern Med. 2001; 16(9): 606-13. pmid: 11556941.

[19] Ibrahim MS, Pang D, Randhawa G, Pappas Y. Development and Validation of a Simple Risk Model for Predicting Metabolic Syndrome (MetS) in Midlife: A Cohort Study. Diabetes Metab Syndr Obes. 2022; 15: 1051-1075. pmid: 35418767.

[20] Barroso WKS, Rodrigues CIS, Bortolotto LA, Mota-Gomes MA, Brandão AA, Feitosa ADM, et al. Diretrizes Brasileiras de Hipertensão Arterial – 2020. Arq. Bras. Cardiol. 2021; 116(3): 516-658. https://doi.org/10.36660/abc.20201238

[21] Heitkemper EM, Mamykina L, Travers J, Smaldone A. Do health information technology self-management interventions improve glycemic control in medically underserved adults with diabetes? A systematic review and meta-analysis. J Am Med Inform Assoc. 2017; 24(5):1024-1035. pmid: 28379397.

	**STUDY PERIOD**							
	Enrollment	Baseline	Weeks or Months after baseline					
TIMEPOINT	Before t = 0	t = 0	Between 1 and 6 weeks	At 7 weeks	At 3 months	At 6 months	At 12 months	After 12 months
ENROLMENT:								
Eligibility Screen	X							
Informed Consent		X						
Application of questionnaires to record demographic and clinical and laboratory data **at baseline**		X						
INTERVENTIONS:								
One-on-one sessions about hypoglycemia, nutrition, physical activity, lifestyle, mental health, and regular use of medication			X					
Orientation session about administering the insulin injection				X				
Measurement of HbA1c, TIR, weight, presence of complications.					X	X	X	
Assessment of medication adherence, health care utilization, and health-related quality of life						X	X	
STATISTICAL ANALYSES					X	X	X	X
