# Supplementary material for: A data-driven approach to manage type 2 diabetes mellitus through digital health: The Klivo Intervention Program protocol (KIPDM)
Source: PLoS One. 2023 Feb 24;18(2):e0281844. doi: 10.1371/journal.pone.0281844 (PMC9956061; doi:10.1371/journal.pone.0281844)
Supplement: S1 Protocol — (PDF) [file pone.0281844.s002.pdf]

TÍTULO DO PROJETO

Protocolo do Programa de Intervenção Klivo: gerenciamento do diabetes mellitus tipo 2 através de uma plataforma digital

Camila Maciel de Oliveira

Investigadora principal

Professora Convidada da Liga de Inovação da PUC-MG (Campus Poços de Caldas).

Klivo LLC, São Paulo, São Paulo, Brasil e Departamento de Medicina Integrativa, Universidade Federal do Paraná, Curitiba, Paraná, Brasil

Luiza Borcony Bolognesi

Orientanda, Sociedade Mineira de Cultura (PUC Minas), Poços de Caldas, Minas Gerais

Poços de Caldas, 2021

VERSÃO 2 DO PROJETO - PÁGINA DE ASSINATURAS

TERMO DE COMPROMISSO, RESPONSABILIDADE, CONFIDENCIALIDADE E QUALIFICAÇÃO DA EQUIPE DA PESQUISA

Nós, autores da pesquisa "**Protocolo do Programa de Intervenção Klivo: gerenciamento do diabetes mellitus tipo 2 através de uma plataforma digital**", solicitamos dispensa do TCLE (Termo de Consentimento Livre e Esclarecido) visto que este será um estudo prospectivo, observacional e transversal, não havendo intervenção ou contato com o paciente. Será realizada a análise de dados disponibilizados por uma empresa parceira (Klivo) que acompanha pacientes diabéticos através de uma plataforma digital. O protocolo desenvolvido e operado pela empresa Klivo encontra-se detalhadamente descrito na sessão metodologia deste projeto, porém ressaltamos que os pesquisadores utilizarão apenas o banco de dados para análises estatísticas e que eles receberão os dados decodificados, de forma que a identidade de cada paciente esteja plenamente preservada. Assim sendo, os pesquisadores asseguram que o caráter anônimo dos pacientes será mantido e que suas identidades serão protegidas. As fichas clínicas ou outros documentos submetidos ao patrocinador, se houverem, não serão identificados pelo nome, mas por um código.

Comprometemo-nos com todos os Termos das Diretrizes e Normas Regulamentadoras de Pesquisa Envolvendo Seres Humanos - Resolução 466/12, 251/97, 292/99, 346/05, 340/04, 404/08, 441/11 e complementares do Conselho Nacional de Saúde e da Agência Nacional de Vigilância Sanitária, incluindo tornar público os resultados desta pesquisa quer sejam eles favoráveis ou não. Comprometemo-nos a informar a este Comitê de Ética qualquer alteração, inclusões ou exclusões de participantes da pesquisa, emendas, interrupções, bem como a conclusão do referido estudo. Comprometemo-nos a enviar, periodicamente e ao final do estudo, relatórios do mesmo ou cópia de comunicação científica.

Autor[1]
Nome: Camila Maciel de Oliveira
Investigadora Principal - http://lattes.cnpq.br/0855677543882534

Autor[2]
Nome: Luiza Borcony Bolognesi
Orientanda

RESUMO

A terapêutica digital, um tipo emergente de terapia médica, é definida como intervenções baseadas em evidências por meio de programas de software qualificados que ajudam a prevenir, gerenciar ou tratar doenças crônicas. Essas intervenções têm desempenhado um papel essencial na gestão de condições metabólicas, como o diabetes mellitus tipo 2, que tem alto impacto social e econômico. Este estudo propõe avaliar uma estratégia de saúde digital em fase inicial de implementação.

Métodos

O Programa de Intervenção Klivo é um método intensivo de intervenção de estilo de vida que busca gerenciar o estilo de vida diário de adultos com diabetes mellitus tipo 2, com idade > 18 anos, com hemoglobina glicada (HbA1c) de 7% ou mais. O programa é gratuito para os participantes inscritos em planos de saúde e organizações prestadoras de plataformas de saúde parceiras. Os resultados como HbA1c e tempo na faixa de glicemia-alvo serão avaliados no momento que antecede a fase de intervenção e nos pontos de tempo estipulados. O programa será baseado em um processo de gestão de 12 meses durante o qual os indivíduos serão supervisionados remotamente por enfermeiros a cada 15 dias. Quando forem detectados parâmetros anormais de glicemia, indivíduos e médicos responsáveis serão contatados de acordo com o protocolo proposto. Os dados clínicos e laboratoriais, controle de peso, qualidade de vida, saúde mental, adesão a medicamentos, confiança na autogestão, utilização da assistência à saúde, alfabetização diabética e sofrimento relacionado à doença serão avaliados por meio de questionários eletrônicos validados. O programa incluirá tele-educação através de telefonemas semanais ao longo de seis semanas. Os exames laboratoriais e dados reportados ao telefone serão avaliados na linha de base e 3, 6, 9 e 12 meses após a intervenção, sendo os questionários aplicados no primeiro e último meses.

Resultados esperados

Os achados deste estudo fornecerão uma visão sobre a melhora da saúde de indivíduos com diabetes mellitus tipo 2 e possivelmente indivíduos com outras doenças cardiometabólicas, incluindo hipertensão arterial, dislipidemia e obesidade.

1. INTRODUÇÃO E REVISÃO DE LITERATURA

A terapêutica digital – um tipo emergente de programas de acompanhamento de saúde do indivíduo que está se expandindo globalmente de acordo com a demanda de mercado – é definida como intervenções baseadas em evidências por meio de software qualificados que ajudam a prevenir, a gerenciar e a tratar doenças crônicas [1]. Alguns estudos sugerem que a avaliação remota contínua e o monitoramento diário podem efetivamente refinar o manejo de condições crônicas como diabetes mellitus tipo 2 (DMT2), a qual tem alta prevalência e impacto social e econômico importantes [2,3]. Nesse sentido, os programas digitais voltados às necessidades individuais dos indivíduos têm contribuído para o manejo contínuo dos hábitos cotidianos e para a redução dos custos de saúde atribuídos às doenças metabólicas crônicas [4,5].

Essas tecnologias têm melhorado a conscientização sobre o hábito alimentar e a prática regular de atividade física, otimizando o controle glicêmico e a adesão ao uso de medicamentos, e, consequentemente, reduzindo o alto custo de tratamento do DMT2 [6]. Sabe-se que o avanço clínico de participantes de protocolos como o Programa Livongo para Diabetes reduz os custos médios por indivíduo por mês em US$ 83 [7], o que é crucial se considerarmos a estimativa de que 700 milhões de pessoas terão a doença em 2045 [2]. Portanto, programas que apoiem mudanças no estilo de vida podem desempenhar importante papel na prevenção secundária ou até mesmo primária ao reduzirem o risco de complicações crônicas ou retardarem o diagnóstico da doença [8].

Algumas organizações de saúde têm oferecido diversos programas digitais como uma parte do modelo de cuidados baseados em valor [9]. Há um consenso de que o valor é criado pela viabilização da saúde e não apenas pela prestação de cuidados. Além disso, os profissionais que utilizam tecnologias digitais em saúde se beneficiam de melhorias a curto e longo prazos [10]. Por isso, pretendemos descrever o protocolo de uma estratégia digital de saúde – o Programa de Intervenção Klivo (PIK) – que está em fase inicial de implementação.

2. OBJETIVOS

2.1 Objetivo principal:

Este estudo avaliará os aspectos clínicos e laboratoriais autorreferidos de indivíduos com DMT2 recrutados para a coorte PIK. Além disso, a saúde mental, a adesão aos medicamentos, a confiança na autogestão, a utilização da saúde, a alfabetização diabética e o sofrimento relacionado à doença serão avaliados por meio de questionários validados. Serão avaliados dois desfechos primários: hemoglobina glicada (HbA1c) e o *Time in Range* (TIR, Tempo na Faixa), definido pela porcentagem de tempo que o indivíduo passa com seus níveis de glicemia em uma faixa-alvo de 70 a 180 mg/dL.

2.2 Objetivos específicos:

1. Comparar os valores de HbA1c do indivíduo na linha de base e 3, 6, 9 e 12 meses após a inclusão no PIK.

2. Identificar a porcentagem de Tempo na Faixa (TIR) e, consequentemente, o número de eventos hipoglicêmicos graves ao longo de 12 meses.

2.3 Objetivos secundários:

Avaliação da incidência de complicações secundárias como lesões em retina, renal, cardíaca e cerebrovascular nos meses 1 e 12.

3. HIPÓTESE DA PESQUISA

3.1 Hipótese principal:

Participantes do PIK deverão apresentar melhora em HbA1c e TIR ao longo da intervenção se comparado aos valores de base.

3.2 Hipóteses secundárias:

Não há

3.3 Variáveis da pesquisa:

- variável(is) dependente(s) (desfechos): HbA1c e Time in Range (TIR, porcentagem de tempo com níveis de glicemia ≥70 e ≤180 mg/dL)

- variáveis independentes (causas): não se aplica

- variáveis interferentes: não se aplica

- variáveis de controle: não se aplica

4. CASUÍSTICA E MÉTODO

4.1 Classificação do tipo de pesquisa e desenho do estudo:

Estudo prospectivo, observacional e transversal.

4.2 População e amostra:

Todos os indivíduos inscritos no PIK serão considerados para a coorte, exceto aqueles que se recusarem a compartilhar seus dados ou perderem o acompanhamento. Serão inscritos no estudo indivíduos de ambos os sexos e idade ≥ 18 anos. O objetivo é recrutar pelo menos 100 pacientes.

4.3 Critérios de inclusão e exclusão

Critérios de inclusão

1. Diagnóstico de DMT2 no prontuário eletrônico de seguradoras (HbA1c 7% ou superior; idade ≥ 18 anos).

2. Disponibilidade para receber telefonemas e mensagens para monitoramento da doença e para tele-educação.

3. Disposição para usar os dispositivos de monitoramento padrão (glucosímetro), sincronizados com o sistema de telemonitoramento de acordo com o protocolo proposto, durante o período de estudo de 12 meses.

Critérios de exclusão

1. Comprometimento cognitivo baseado em diagnóstico de demência ou prejuízo cognitivo leve relatado nos prontuários.

2. Resistência autodeclarada em receber telefonemas ou mensagens para o manejo da doença ou perda do acompanhamento.

3. Condição pré-existente: doença renal crônica (estágio 5); indivíduos com qualquer doença em estágio terminal com prognóstico de vida inferior a dois anos; ou mulheres grávidas.

4.4 Plano de recrutamento e Processo de aplicação do TCLE:

Os membros da equipe dos planos de saúde parceiros da startup Klivo serão responsáveis pela pré-triagem de indivíduos diagnosticados com DMT2. Após esta fase, os critérios de elegibilidade serão utilizados. Os potenciais indivíduos que preencherem os critérios de elegibilidade serão contatados pela equipe PIK por telefone para a obtenção do endosso verbal do indivíduo sobre a utilização de seus

dados para fins de análises estatísticas. A assinatura do TCLE pelos participantes será dispensada, mas o TCLE será lido antes do início da pesquisa, durante a própria ligação telefônica e, em seguida, uma cópia do TCLE será enviada ao participante.

4.5 Cenário de realização do estudo, período de coleta de dados:

Klivo é uma startup brasileira fundada em 2020 e certificada pela Sociedade Brasileira de Diabetes (SBC) em primeiro de setembro de 2021. O PIK tem como objetivo acompanhar indivíduos com condições crônicas no autocuidado ao longo do tempo. Seus principais parceiros são as seguradoras de saúde no Brasil. A equipe Klivo apoia os indivíduos na obtenção de resultados consistentes em relação a condições metabólicas e na melhoria de qualidade de vida. Esta plataforma digital facilitará a coleta de dados relacionados à saúde de um indivíduo, analisará dados para avaliar condições clínicas ou pré-clínicas, além do gerenciamento personalizado da jornada do indivíduo ao longo de 12 meses.

4.6 Fonte do material de pesquisa e Fases do desenvolvimento da pesquisa:

Este será um estudo prospectivo, observacional e transversal para a análise de dados de um banco de informações de uma empresa parceira (Klivo) que acompanha pacientes diabéticos através de uma plataforma digital. Os pesquisadores receberão os dados decodificados, de forma que a identidade de cada paciente esteja plenamente preservada.

O protocolo desenvolvido e operado pela empresa Klivo segue detalhado abaixo:

Os membros da equipe dos planos de saúde parceiros da startup Klivo serão responsáveis pela pré-triagem de indivíduos diagnosticados com DMT2. Após esta fase, os critérios de elegibilidade serão utilizados. Os potenciais indivíduos que preencherem os critérios de elegibilidade serão contatados pela equipe Klivo por telefone para a obtenção do endosso verbal do indivíduo sobre a utilização de seus dados para fins de análises estatísticas. A assinatura do TCLE pelos participantes será dispensada, mas o TCLE será lido antes do início da pesquisa, durante a própria ligação telefônica e, em seguida, uma cópia do TCLE será enviada ao participante. Em seguida, a equipe administrará questionários padronizados, remotamente via ligação telefônica, para registrar os dados demográficos, clínicos e laboratoriais do indivíduo. Durante 12 meses, os enfermeiros supervisionarão remotamente os indivíduos a cada 15 dias, através de um processo de gestão. Este método é um programa intensivo de intervenção de estilo de vida adaptado dos Padrões Nacionais de Educação e Suporte de Autogestão do Diabetes [11]. Via ligação telefônica, a equipe adotará questionário padronizado (Consórcio Internacional de Medição de Resultados em Saúde [12]) para registrar dados sobre características demográficas do indivíduo, histórico médico e fatores de risco ambiental. A pressão sanguínea e os parâmetros antropométricos serão baseados em valores autorreferenciados durante o contato telefônico. Glicemia,

HbA1c, colesterol total, triglicérides e lipoproteínas (de alta e baixa densidade) serão medidos por técnicas padrão no laboratório habitual dos indivíduos, após jejum ≥8 h, a cada 3 meses. As escalas de Morisky Green [13], PAID [14], WHO-5 [15] e PHQ-9 [16] também serão aplicadas. O programa incluirá tele-educação para orientação individual através de telefonemas semanais ao longo de 6 semanas (1. Introdução. Questionário. Hipoglicemia; 2. Questionários. Habilidades de resolução de problemas e abordagem IDEA (identificar problema, definir possíveis soluções, avaliar soluções, agir na melhor solução). Definição de plano individualizado para sessões intervencionistas subsequentes; 3. Nutrição e atividade física; 4. Medicamentos e relação entre horário de uso e de refeições; 5. Saúde emocional, tabagismo e álcool; 6. Importância de hábitos saudáveis e controle da glicemia a longo prazo). Indivíduos em insulinoterapia terão sessão adicional sobre administração da injeção. Além de interfaces tradicionais (chamadas telefônicas, e-mails, mensagens de texto e web), o PIK incluirá aplicativos para smartphones e dispositivos vestíveis (klivo.com). Indivíduos usarão dispositivos padronizados habilitados para Bluetooth para transmitir glicemia capilar. Perante parâmetros anormais de glicemia, indivíduos e médicos responsáveis serão contatados através de chamada telefônica (glicemia capilar < 54 mg/dL ou > 450 mg/dL) ou mensagem de texto (glicemia capilar entre 55 e 70 g/dL ou entre 350 e 449 mg/dL). Indivíduos serão monitorados a cada 15 minutos para garantir reversão da hipo ou hiperglicemia. Se necessidades específicas forem detectadas, enfermeiros sugerirão ligações adicionais com equipe multidisciplinar (psicólogos, nutricionistas e educadores físicos). Se o indivíduo apresentar ≥3 episódios de hipoglicemia, o médico receberá um e-mail com um relatório sobre o controle glicêmico do indivíduo. Informações coletadas serão criptografadas e armazenadas em sigilo para uso exclusivo do Núcleo de Pesquisas Klivo. No evento de distribuição para membros da equipe ou terceiros para fins de análises estatísticas, os dados dos indivíduos serão descaracterizados para a preservação da privacidade.

Além das interfaces tradicionais, como chamadas telefônicas, e-mails, mensagens de texto e web, a Klivo incluirá estratégias baseadas em tecnologia, como aplicativos para smartphones e dispositivos vestíveis (klivo.com). No entanto, o telemonitoramento ainda será necessário, principalmente para a população idosa [17]. Os indivíduos usarão dispositivos padronizados habilitados para Bluetooth para a transmissão da glicemia capilar.

4.7 Medidas de proteção ou minimização de qualquer risco eventual ao participante da pesquisa

O programa envolve ações educativas em saúde e monitoramento (especialmente controle glicêmico).

Quanto a riscos relacionados ao diagnóstico da doença (hipoglicemia ou hiperglicemia, por exemplo), medidas serão tomadas para mitigar tais eventos. Uma chamada

telefônica será feita ou uma mensagem de texto será enviada se forem detectados parâmetros anormais de glicemia. Chamadas telefônicas se a glicemia capilar < 54 mg/dL ou > 450 mg/dL e mensagem de texto se entre 55 e 70 g/dL ou entre 350 e 449 mg/dL. Os indivíduos serão monitorados a cada 15 minutos para garantir que a hipo ou a hiperglicemia sejam revertidas.

Se forem detectadas necessidades específicas, os enfermeiros sugerirão ligações adicionais com equipe multidisciplinar, incluindo psicólogos, nutricionistas e educadores físicos.

Se o indivíduo apresentar três ou mais episódios de hipoglicemia, o médico receberá um e-mail com um relatório sobre o controle glicêmico do indivíduo.

4.8 Detalhamento do instrumento de coleta de dados

Estas atividades serão desempenhadas pela empresa parceira (Klivo):

A intervenção começará com uma sessão de orientação de 60 minutos sobre o programa. O educador de diabetes ligará para o indivíduo no horário previamente agendado.

O PIK incluirá tele-educação por meio de telefonemas semanais ao longo de seis semanas em sessões individuais sobre hipoglicemia, nutrição, atividade física, estilo de vida, saúde mental e uso regular de medicamentos conforme descrito abaixo.

Sessões

1. Introdução. Questionário. Orientação individual sobre hipoglicemia.

2. Questionários. Habilidades de resolução de problemas e abordagem IDEA (identificar o problema, definir possíveis soluções, avaliar as soluções, agir na melhor solução). Definição de um plano individualizado para as sessões intervencionistas subsequentes.

3. Orientação individual sobre nutrição e atividade física.

4. Orientação individual sobre medicamentos em uso, especialmente sobre a relação entre o horário de uso do hipoglicemiante e de realização das refeições.

5. Orientação individual sobre saúde emocional, tabagismo e álcool.

6. Orientação individual sobre a importância da manutenção de hábitos saudáveis e controle da glicemia a longo prazo.

Os indivíduos em insulinoterapia terão uma sessão adicional para orientação sobre a administração da injeção.

Monitorização

As enfermeiras farão telefonemas para cada indivíduo a cada quinze dias ao longo de doze meses para apoiar a autogestão contínua do DMT2. Todos os indivíduos continuarão recebendo tratamento de seus médicos regulares durante o período de 12 meses.

Além disso, dependendo da gravidade da situação, uma chamada telefônica será feita ou uma mensagem de texto será enviada se forem detectados parâmetros anormais de glicemia. Chamadas telefônicas se a glicemia capilar < 54 mg/dL ou > 450 mg/dL e mensagem de texto se entre 55 e 70 g/dL ou entre 350 e 449 mg/dL. Os indivíduos serão monitorados a cada 15 minutos para garantir que a hipo ou a hiperglicemia sejam revertidas.

Se forem detectadas necessidades específicas, os enfermeiros sugerirão ligações adicionais com equipe multidisciplinar, incluindo psicólogos, nutricionistas e educadores físicos.

Contato com médicos de referência

Se o indivíduo apresentar três ou mais episódios de hipoglicemia, o médico receberá um e-mail com um relatório sobre o controle glicêmico do indivíduo.

Medições clínicas e laboratoriais

Questionário

Para obter informações relacionadas às características demográficas do indivíduo, ao histórico médico e aos fatores de risco ambiental, cada indivíduo responderá a perguntas durante uma ligação telefônica quando o enfermeiro preencherá um questionamento eletrônico projetado de acordo com os critérios do ICHOM [12]. Além disso, serão coletadas e definidas informações sobre atividade física (tempo por semana), estado de tabagismo e consumo de álcool (quantidade e frequência por semana) de acordo com os critérios do ICHOM [12].

Para verificar a adesão aos medicamentos, os dados serão coletados através de um instrumento padronizado chamado escala Morisky Green [13]. A relação entre indivíduo e DMT2 será avaliada pelo questionário Áreas de Problemas em Diabetes (PAID) [14]. O instrumento índice de bem-estar da OMS (WHO-5) será utilizado para avaliar o bem-estar psicológico [15]. O estado de depressão será estimado pelo Questionário de Saúde do Indivíduo (PHQ-9) [16] e encaminhamentos estratégicos serão sugeridos de acordo com o escore.

Medição da pressão arterial

As pressões sanguíneas sistólicas e diastólicas serão baseadas em valores relatados pelo indivíduo durante o contato telefônico (autorreferenciados).

Parâmetros antropométricos

Os indivíduos relatarão os parâmetros antropométricos durante o contato telefônico. Para medir a circunferência da cintura (CC), os indivíduos serão instruídos a colocar uma fita métrica ao redor de seu corpo, ao nível do umbigo. O aumento do CC é definido como > 88 cm para as mulheres e > 102 cm para os homens.

O índice de massa corporal (IMC) será calculado como o peso corporal (kg) dividido pelo quadrado da altura (m^2). O sobrepeso será definido como IMC ≥ 25 kg/m^2 e < 30 kg/m^2, e a obesidade será definida como IMC ≥ 30 kg/m^2.

Análise bioquímica

Valores de glicemia, HbA1c, colesterol total, triglicérides e lipoproteínas como lipoproteína de alta densidade (HDL-c) e lipoproteína de baixa densidade (LDL-c) serão medidas por técnicas padrão no laboratório habitual dos indivíduos, após jejum de pelo menos 8 horas, a cada três meses.

Diagnóstico da doença

A pressão arterial sistólica (SBP) ≥140 mmHg ou pressão arterial diastólica (DBP) ≥90 mmHg (medidas no consultório médico ou em casa) ou uso de medicamentos anti-hipertensivos serão os parâmetros considerados para o diagnóstico de hipertensão arterial [18]. O DMT2 será definido pela presença de glicose em jejum ≥ 126mg/dL ou uso de drogas hipoglicemiantes. A dislipidemia será definida pelo uso de drogas.

4.9 Análise estatística

<u>Estas atividades serão desempenhadas pela investigadora Profa. Dra. Camila Maciel de Oliveira e orientanda:</u>

A análise será realizada a partir da comparação de parâmetros pré- e pós-intervenção para os indivíduos selecionados no PIK.

Para todas as medidas, as características clínicas e laboratoriais serão avaliadas por estatísticas descritivas. As variáveis categóricas serão expressas em percentuais e variáveis contínuas como média ± DP ou mediana (intervalo interquartil). O teste de Kolmogorov-Smirnov será usado para verificar a normalidade dos dados. As características dos indivíduos nos diferentes grupos (definidos de acordo com características clínicas) serão avaliadas por teste-t ou teste de classificação Wilcoxon (variáveis contínuas) ou teste qui-quadrado de Pearson (variáveis categóricas).

Regressão logística será utilizada para avaliar a associação entre variáveis independentes e diabetes. As análises serão necessariamente ajustadas para idade e sexo. As curvas ROC serão realizadas para avaliar o desempenho dos modelos sugeridos e que tenham plausabilidade científica, e a área sob a curva (AUC) será utilizada para medir o poder discriminatório das variáveis explicativas identificadas para o DMT2. A análise estatística será realizada utilizando-se a versão 1.3.1093do software RStudio. O nível de significância será fixado em 5%.

Subgrupos a serem criados *a posteriori* serão pautados em condições clínicas (por exemplo, grupo I e grupo II) descritas da seguinte forma:

Grupo I: Indivíduos sem alterações ou apresentando apenas retinopatia diabética não proliferativa leve sem qualquer envolvimento macular; doença renal crônica até o estágio 3a (eGFR $\geq$ 45 mL/min/1,73 m^2); indivíduos sem doenças macrovasculares conhecidas.

Grupo II: Indivíduos com patologias da retina, incluindo retinopatia diabética proliferativa (moderada a grave) ou outras doenças da retina ou macular; ou doença renal crônica estágio 3b ou 4; ou doença vascular periférica, coronariana ou cerebrovascular conhecida.

5. DESFECHOS

Resultados esperados sob o ponto de vista acadêmico:

Participantes do PIK deverão apresentar melhora em HbA1c e TIR ao longo da intervenção se comparado aos valores de base.

6. RISCOS E BENEFÍCIOS

6.1 Riscos: O programa envolve ações educativas em saúde e monitoramento (especialmente controle glicêmico). Há riscos relacionados ao diagnóstico da doença (hipoglicemia ou hiperglicemia, por exemplo) e às medidas tomadas para mitigar tais eventos. Há também riscos envolvendo manuseio de dados (vazamento de dados, ataques hackers, riscos à segurança e privacidade, por exemplo).

6.2 Previsão de ressarcimento de gastos aos participantes da pesquisa:

Não se aplica

6.3 Benefícios:

Os pacientes receberão informações acerca da patologia de base (diabetes mellitus) e fatores asssociados. Alertas imediatos serão enviadas aos pacientes, quando valores glicêmicos anormais.

7. CRONOGRAMA

	2022										2023	
	M	A	M	J	J	A	S	O	N	D	J	F
Especificação dos objetivos e hipóteses.												
Pesquisa Bibliográfica.												
Aprovação do projeto por agências financiadoras												
Aprovação do projeto CEP												
Operacionalização conceitos e variáveis.												
Coleta de dados.												
Realização dos testes laboratoriais (se aplicável)	NÃO APLICÁVEL											
Compilação dos dados.												
Análise dos resultados.												
Trat. estatístico e análise dos dados												
Discussão e Conclusão final.												
Defesa/apresentação												
Publicação em periódicos científicos.												

A coleta de dados na linha de base (tempo 0) e intervenção terão início em 03/03/2022. Dados serão novamente coletados 3, 6, 9 e 12 meses após o início do protocolo.

8. RECURSOS NECESSÁRIOS E CUSTOS DO PROJETO

8.1 Equipe executora:

Profa. Dra. Camila Maciel de Oliveira e Luiza Borcony Bolognesi

8.2 Suprimentos e equipamentos:

não se aplica

8.3 Riscos associados aos recursos necessários:

não se aplica

8.4 Orçamento detalhado:

Empresa financiadora proverá R$100.000,00 para custear o projeto

8.5 Financiadores: KLIVO LICENCIAMENTO LTDA (CNPJ 15.996.337/0001-85) (ver declaração de ciência e concordância assinada anexada ao projeto na Plataforma Brasil)

9. REFERÊNCIAS

[1] Hong JS, Wasden C, Han DH. Introdução da terapêutica digital. Programas de Métodos computacionais Biomed. 2021; 209: 106319. pmid: 34364181.

[2] Saeedi P, Petersohn I, Salpea P, Malanda B, Karuranga S, Unwin N, et al; Comitê atlas de diabetes do IDF. Estimativas de prevalência global e regional de diabetes para 2019 e projeções para 2030 e 2045: Resultados do Atlas da Federação Internacional de Diabetes, 9ª edição. Diabetes Res Clin Pract. 2019; 157: 107843. pmid: 31518657.

[3] Rhee SY, Kim C, Shin DW, Steinhubl SR. Presente e futuro da saúde digital em diabetes e doença metabólica. Diabetes Metab J. 2020; 44(6): 819-27. pmid: 33389956.

[4] Thorpe K, Toles A, Shah B, Schneider J, Bravata MD. Redução associada à perda de peso nos gastos com assistência médica para indivíduos com seguro comercial com condições crônicas. J Occup Environ Med. 2021; 16 de junho. doi: 10.1097/jom.0000000000002296. Epub à frente da impressão. pmid: 34138824.

[5] Huckfeld PJ, Frenier C, Pajewski NM, Espeland M, Peters A, Casanova R, et al. Associações de intervenção intensiva do estilo de vida no diabetes tipo 2 com uso de cuidados de saúde, gastos e incapacidade: estudo auxiliar do estudo look AHEAD. JAMA Netw Open. 2020; 3(11): e2025488. pmid: 33231638.

[6] Downing J, Bollyky J, Schneider J. Uso de um medidor de glicose conectado e treinamento certificado de educador de diabetes para diminuir a probabilidade de excursões anormais de glicose: o Programa Livongo para Diabetes. J Med Internet Res. 2017; 19(7): e234. pmid: 28698167.

[7] Bollyky JB, Bravata D, Yang J, Williamson M, Schneider J. Coaching de estilo de vida remoto, além de um medidor de glicose conectado com suporte certificado de educador de diabetes melhora a glicose e a perda de peso para pessoas com diabetes tipo 2. J Diabetes Res. 2018: 3961730. pmid: 29888288.

[8] Goh KLS, Lee CS, Koh CHG, Ling NL, Ang SB, Oh C. Avaliando a eficácia e a utilidade de um novo sistema de telemonitoramento culturalmente adaptado para melhorar o controle glicêmico dos asiáticos com diabetes mellitus tipo 2: um protocolo de estudo de método misto. Ensaios. 2021; 22(1): 305. pmid: 33902656.

[9] Porter ME, Teisberg EO. Redefinição da saúde - criando concorrência baseada em valor em resultados. Boston: Harvard Business School Press; 2006.

[10] Kaufman N, Khurana I. Utilizando tecnologia digital de saúde para prevenir e tratar o diabetes. Diabetes Technol Ther. 2016; 18 Suppl 1 (Suppl 1): S56-S68. pmid: 26836430.

[11] Beck J, Greenwood DA, Blanton L, Bollinger ST, Butcher MK, Condon JE, et al. 2017 National standards for diabetes self-management education and support. Diabetes Care. 2017; 40(10):1409-19. https://doi.org/10.2337/dci17-0025.

[12] Nano J, Carinci F, Okunade O, Whittaker S, Walbaum M, Barnard-Kelly, K, et al.; Grupo de Trabalho de Diabetes do Consórcio Internacional de Medição de Resultados em Saúde (ICHOM). Um conjunto padrão de desfechos centrados nas pessoas para diabetes mellitus: resultados de uma abordagem internacional e unificada. Diabet Med. 2020; 37(12): 2009-18. pmid: 3214488.

[13] Morisky DE, Green LW, Levine DM. Concurrent and predictive validity of a self-reported measure of medication adherence. Med Care. 1986; 24(1): 67-74. pmid: 3945130.

[14] Schmitt A, Reimer A, Kulzer B, Haak T, Ehrmann D, Hermanns N. How to assess diabetes distress: comparison of the Problem Areas in Diabetes Scale (PAID) and the Diabetes Distress Scale (DDS). Diabet Med. 2016; 33(6): 835-43. pmid:26287511.

[15] Topp CW, Østergaard SD, Søndergaard S, Bech P. The WHO-5 Well-Being Index: a systematic review of the literature. Psychother Psychosom. 2015; 84(3): 167-76. pmid: 25831962.

[16] Koenke K, Spitzer RL, Williams JBW. The PHQ-9 validity of a brief depression severity measure. J Gen Intern Med. 2001; 16(9): 606-13. pmid: 11556941.

[17] Munshi MN, Meneilly GS, Rodríguez-Mañas L, Close KL, Conlin PR, Cukierman-Yaffe T, et al. Diabetes in ageing: pathways for developing the evidence base for clinical guidance. Lancet Diabetes Endocrinol. 2020; 8(10): 855-67. pmid: 32946822.

[18] Barroso WKS, Rodrigues CIS, Bortolotto LA, Mota-Gomes MA, Brandão AA, Feitosa ADM, et al. Diretrizes Brasileiras de Hipertensão Arterial – 2020. Arq. Bras. Cardiol. 2021; 116(3): 516-658. https://doi.org/10.36660/abc.20201238

ANEXO DO MODELO DE TCLE/ QUESTIONÁRIOS/ E/OU INSTRUMENTOS DE COLETA DE DADOS

Cópias dos modelos de TCLE, questionários e instrumentos de coleta de dados foram adicionados à Plataforma Brasil.
