# Supplementary material for: A data-driven approach to manage type 2 diabetes mellitus through digital health: The Klivo Intervention Program protocol (KIPDM)
Source: PLoS One. 2023 Feb 24;18(2):e0281844. doi: 10.1371/journal.pone.0281844 (PMC9956061; doi:10.1371/journal.pone.0281844)
Supplement: S2 Protocol — (PDF) [file pone.0281844.s003.pdf]

CYNTHIA MARIA DE CAMPOS PRADO MANSO
TRADUTORA PÚBLICA E INTÉRPRETE COMERCIAL – INGLÊS
Matrícula JUCESP nº 792
C.C.M. 9821401 RG 20.907.343-3-SSP-SP CPF 180.988.858-10
Rua Campos Salles nº 1150 apto. 11 – Centro – CEP 14015-110
RIBEIRÃO PRETO – SP – TEL (16) 3630-8603 – CEL (16) 99993-3932

To whom it may concern, I declare that I am a Sworn Translator and Commercial Interpreter duly sworn by the Board of Trade of the State of São Paulo (JUCESP) - Federative Republic of Brazil and I certify in good faith that I am fluent in both the English and Portuguese languages; that on this date an original document in the Portuguese language identified as PONTIFÍCIA UNIVERSIDADE CATÓLICA DE MINAS GERAIS PRÓ-REITORIA DE PESQUISA E DE PÓS-GRADUAÇÃO COMITÊ DE ÉTICA EM PESQUISA – CEP TÍTULO DO PROJETO PROTOCOLO DO PROGRAMA DE INTERVENÇÃO KLIVO: GERENCIAMENTO DO DIABETES MELLITUS TIPO 2 ATRAVÉS DE UMA PLATAFORMA DIGITAL was presented to me by its bearer for the sole purpose of translating said document into English; and that the text attached below is a true and complete translation of said document to the best of my knowledge and ability:

[logo on the top left]: PUC Minas, The Pontifical Catholic University of Minas Gerais

THE PONTIFICAL CATHOLIC UNIVERSITY OF MINAS GERAIS
Office of the Provost of Research and Postgraduate Studies
Research Ethics Committee (REC, or CEP (abbreviation in Portuguese))

TITLE OF PROJECT

The Klivo Intervention Program protocol: management of type 2 diabetes mellitus through a digital platform

Camila Maciel de Oliveira

Main investigator

Invited Professor of the Health Innovation Program of PUC Minas (campus in the city of Poços de Caldas, state of Minas Gerais, Brazil)

Klivo LLC, city of São Paulo, state of São Paulo, Brazil

Department of Integrative Medicine, Federal University of Paraná, city of Curitiba, state of Paraná, Brazil)

Luiza Borcony Bolognese

Advisee

Sociedade Mineira de Cultura (PUC Minas), campus in the city of Poços de Caldas, state of Minas Gerais, Brazil

Poços de Caldas, 2021

PAGE WITH SIGNATURES

TERM OF COMMITMENT, LIABILITY, CONFIDENTIALITY AND RESEARCH GROUP QUALIFICATION

We, authors of the research "**The Klivo Intervention Program protocol: management of type 2 diabetes mellitus through a digital platform**", request that the need for signing a written free informed consent be dismissed given that this will be a prospective, observational, and transversal study that will not imply contact between the authors of the research and the participants, and that the authors of the research will not engage in any intervention with the participants. The authors of the research will only analyze the data made available by a partner startup (Klivo), which monitors patients with diabetes mellitus through a digital platform. The protocol developed and operated by the startup Klivo is detailed in the methodology section of this project, but we would like to highlight that the authors of the research will only use the database for statistical analyses, and that the authors will receive de-identified data, while the participants' identity will remain totally anonymous. In other words, the authors of the research assure the Research Ethics Committee that the participants' identity will remain anonymous and protected. If any clinical records or other documents are submitted by the partner startup, they will be identified by a code and not by the participant's name.

We agree to all the provisions listed in the Terms of the Regulating Guidelines and Norms of Research Involving Humans – Resolution 466/12, 251/97, 292/99, 346/05, 340/04, 404/08, 441/11 of the Brazilian National Health Council and National Health Surveillance Agency, and we will make the results of this research public no matter whether they are favorable or not. We will inform this Research Ethics Committee of any alterations, participants' inclusion or exclusion, amendments, interruptions, and conclusion of the study. We will send reports or copies of scientific communications to the Research Ethics Committee on a regular basis and at the end of the study.

With the signatures of the Authors Camila Maciel de Oliveira, Main Investigator, http://lattes.cnpq.br/0855677543882534, and Luiza Borcony Bolognesi, Advisee.

ABSTRACT

Digital therapeutics, an emerging type of medical approach, is defined as evidence-based therapeutic interventions through qualified software programs that help prevent, manage, or treat chronic diseases. These interventions have played an essential role in the management of metabolic conditions, such as type 2 diabetes mellitus, which has high social and economic burden. This study proposes evaluating a digital health strategy in the initial phase of implementation.

Methods

The Klivo Intervention Program is an intensive lifestyle intervention method that seeks to improve the daily lifestyle of adults with type 2 diabetes mellitus, aged ≥ 18 years, with glycated hemoglobin (HbA1c) of 7% or higher. The program is free for patients that are users of partner health plans and healthcare provider organizations. HbA1c and time in target blood glucose range will be evaluated at baseline and stipulated time points. The program will be based on a 12-month management process during which participants will be remotely supervised by nurses every fifteen days. When abnormal glucose levels are detected, the participant and their medical doctor will be contacted according to an established protocol. Clinical and laboratory data, weight control, quality of life, mental health, medication adherence, confidence in self-management, healthcare utilization, diabetic literacy and related distress will be evaluated through validated electronic questionnaires. The program will include tele-education via phone calls along the first six weeks. The laboratorial data and data reported over the phone will be assessed at baseline and 3, 6, 9, and 12 months after intervention; the questionnaires will be applied in the first and last months.

Expected outcomes

Findings from this study will provide insights into the health improvement of individuals with type 2 diabetes mellitus and possibly of individuals with other cardiometabolic conditions, including hypertension, dyslipidemia, and obesity.

1. INTRODUCTION AND REVIEW OF THE LITERATURE

Digital therapeutics – an emerging type of medical approach that is expanding globally with market demand – is defined as evidence-based therapeutic interventions through qualified software programs that help prevent, manage, and treat chronic diseases [1]. Some studies have suggested that continuous remote evaluation and daily monitoring can effectively refine the management of chronic conditions such as type 2 diabetes mellitus (T2DM), which has a high prevalence and social and economic burden [2,3]. In this sense, digital therapeutics products addressed to individual needs have contributed to ongoing management of daily life habits and to lowering healthcare costs attributed to chronic metabolic diseases [4,5].

These technologies have improved awareness about diet and regular exercise, optimized glycemic control, and ensured adherence to medication use, consequently lowering the high cost of treating T2DM [6]. Indeed, clinical advancement of participants in protocols such as the Livongo for Diabetes Program has lowered average costs per patient per month by $83 [7], which is crucial if we consider the estimation that 700 million people will be living with diabetes in 2045 [2]. Therefore, programs that support lifestyle changes can perform secondary prevention by reducing the risk of chronic complications or even primary prevention by delaying disease diagnosis [8].

Some healthcare organizations have offered digital programs as part of the value-based care model [9]. There is a consensus that value is created by enabling health and not by just delivering care. Furthermore, professionals who use digital health technologies benefit from short-term and long-term health improvements [10]. Therefore, here we intend to describe the use of a digital health strategy – the Klivo Intervention Program (KIP) – which is in the initial phase of implementation.

2. OBJECTIVES

2.1 Main objective:
This study will evaluate the self-reported clinical and laboratorial aspects of patients with T2DM recruited for the KIP cohort. Additionally, mental health, medication adherence, confidence in self-management, healthcare utilization, diabetic literacy, and related distress will be evaluated by using validated questionnaires. Two primary outcomes will be evaluated: glycated hemoglobin (HbA1c) and time in range (TIR), defined by the percentage of time an individual remains with blood glucose levels in a target range of 70 to 180 mg/dL.

2.2 Specific objectives:
1. To compare the participant’s HbA1c values at baseline and 3, 6, 9, and 12 months after inclusion in KIP.
2. To identify the percentage of time in range (TIR) and, hence, the number of severe hypoglycemic events over 12 months.

2.3 Secondary objectives:
To evaluate the incidence of secondary complications like retinal, renal, cardiac, and cerebrovascular lesions 1 month and 12 months after inclusion in KIP.

3. RESEARCH HYPOTHESES

3.1 Main hypothesis:
KIP participants should present improved HbA1c and TIR along the intervention as compared to baseline values.

3.2 Secondary hypothesis:
None

3.3 Research variables:
- dependent variable(s): (outcomes): HbA1c and time in range (TIR, percentage of time an individual remains with blood glucose levels in a target range of 70 to 180 mg/dL)
- independent variables (causes): non-applicable
- interfering variables (causes): non-applicable
- control variables (causes): non-applicable

4. STUDY OF CASES AND METHOD

4.1 Study type and design:
Prospective, observational, and transversal study.

4.2 Population and sampling:
All the patients enrolled for KIP will be considered for the cohort, except patients who refuse to share their data or lose follow-up. Patients of both sexes and aged ≥ 18 years will be included. We aim to recruit at least 100 participants.

4.3 Inclusion and exclusion criteria:
Inclusion criteria
1. Diagnosis of T2DM in the electronic medical record of private health insurance companies (HbA1c reading 7% or higher; age ≥ 18 years).
2. Willingness to receive phone calls and messages for monitoring the disease and for tele-education.
3. Willingness to use the standard monitoring devices (glucometer), synchronized with the telemonitoring system according to the study protocol throughout the 12-month study period.

Exclusion criteria
1. Cognitive impairment based on a diagnosis of dementia or mild cognitive impairment reported in the medical records.
2. Self-declared reluctance to receive phone calls or messages for disease management.
3. Pre-existing condition: chronic kidney disease stage 5; patients with any end-stage disease with a life prognosis of fewer than two years; or pregnant women.

4.4 Recruitment plan and Process for obtaining consent:
The team members of private health insurance companies partnered with the Klivo startup will pre-screen patients diagnosed with T2DM. After this stage, the eligibility criteria will be used. Patients meeting the eligibility criteria will be contacted by a Klivo team member via a phone call, during which they will be asked for verbal authorization to share their data for statistical analysis. Participants will be dismissed from signing a written free informed consent, but the terms of the free informed consent will be read to them during the phone call, before the starch of the research, and a written copy of the terms will be sent to them.

4.5 Study setting, data collection timeframe:
Klivo is a Brazilian startup founded in 2020 and certified by the Brazilian Society of Diabetes in September 2021. KIP seeks to follow up individuals with chronic conditions in their selfcare along time. Its main partners are health insurance companies in Brazil. The Klivo team supports individuals in obtaining consistent results in metabolic conditions and improving their quality of life. This digital platform will facilitate data collection related to an individual's health, analyze data to evaluate clinical or pre-clinical conditions, and will provide personalized management of the individual's journey along 12 months.

4.6 Source of research material and Research development phases:
This is a prospective, observational, and transversal study that will analyze data from a database of a partner startup (Klivo), which follows up patients with diabetes mellitus through a digital platform. The authors of this research will receive encrypted data, while the participants' identity will remain totally anonymous.

The protocol developed and operated by the startup Klivo is detailed below:

The private health insurance team members will pre-screen patients diagnosed with T2DM for eligibility as described below and advise a Klivo team member to approach the identified patients for registration in KIP. The adopted eligibility criteria are as follows.

The team members of private health insurance companies partnered with the Klivo startup will pre-screen patients diagnosed with T2DM. After this stage, the eligibility criteria will be used. Patients meeting the eligibility criteria will be contacted by a Klivo team member via a phone call, during which they will be asked for verbal authorization to share their data for statistical analysis. Participants will be dismissed from signing a written free informed consent, but the terms of the free informed consent will be read to them during the phone call, before the starch of the research, and a written copy of the term will be sent to them. Next, the team will remotely apply standardized questionnaires via phone calls, to record the participants' demographic, clinical, and laboratorial data. For 12 months, nurses will remotely supervise participants through a management process. This method is an intensive lifestyle intervention program adapted from the National Standards for Diabetes Self-Management Education and Support [11]. Via a phone call, the team will adopt a standardized questionnaire (The International Consortium for Health Outcomes Measurement [12]) to record data about the participants' demographic characteristics, medical history, and environmental risk factors. Blood pressure and anthropometric parameters will be based on self-reported values obtained during the phone call. Blood glucose, HbA1c, total cholesterol, triglycerides, and lipoprotein fractions as high-density lipoprotein (HDL-c) and low-density lipoprotein (LDL-c) will be measured by standard techniques in the participants' usual laboratory, after fasting for at least eight hours, every three months. The scales Morisky Green [13], PAID [14], WHO-5 [15], and PHQ-9 [16] will also be applied. The program will include tele-education via weekly phone calls over six weeks in one-on-one orientation sessions (1. Introduction. Questionnaires. One-on-one orientation about hypoglycemia. 2. Questionnaires. Problem-solving skills and IDEA approach (Identify the problem, Define possible solutions, Evaluate the solutions, Act on the best solution). Definition of an individualized plan for the subsequent interventional sessions. 3. Nutrition and physical activity. 4. Medications and relation between administration time and meals. 5. Emotional health, smoking, and alcohol. 6. Importance of healthy habits and glucose control in the long term. Participants that require insulin therapy will have an additional session about administering the injection. Besides traditional interfaces (phone calls, e-mails, text messages, and the Web), KIP will include smartphone applications and wearable devices (klivo.com). Participants will use standardized Bluetooth-enabled devices to transmit their capillary glucose. When abnormal glucose levels are detected, the participant and their medical doctors will be contacted via a phone call (capillary glucose < 54 mg/dL or > 450 mg/dL) or text message (capillary glucose between 55 and 70 g/dL or 350 and 449 mg/dL). The participant will be monitored every 15 minutes to guarantee that hypo or hyperglycemia is reverted. If specific needs are detected, nurses will suggest additional scheduled phone calls with a multidisciplinary team (psychologists, nutritionists, and physical educators). If the participant presents ≥ 3 episodes of hypoglycemia, their medical doctor will receive an e-mail with a report about the participant's glycemic control. Collected information will be encrypted, stored, and kept confidential for exclusive use of the Klivo Research Center. In case of distribution to team members or third parties for the purpose of statistical analyses, participants' data will be de-identified to ensure privacy.

Besides traditional interfaces (phone calls, e-mails, text messages, and the Web), Klivo will include smartphone applications and wearable devices (klivo.com). However, telemonitoring

will still be needed, mainly for the elderly population [17]. Participants will use standardized Bluetooth-enabled devices to transmit their capillary glucose.

4.7 Protective measures or minimization of any risks for the participants
The program involves educational action in health and monitoring (mainly glucose level control).

Concerning the risks associated with disease diagnosis (for example, hypo or hyperglycemia), measures will be taken to mitigate such events. A phone call will be made, or a text message will be sent if abnormal glucose parameters are detected. Phone calls will be made if capillary glucose is < 54 mg/dL or > 450 mg/dL, and text messages will be sent if capillary glucose is between 55 and 70 g/dL or 350 and 449 mg/dL. The participant will be monitored every 15 minutes to guarantee that hypo or hyperglycemia is reverted.

If specific needs are detected, nurses will suggest additional scheduled phone calls with a multidisciplinary team (psychologists, nutritionists, and physical educators).

If the participant presents three or more episodes of hypoglycemia, their medical doctor will receive an e-mail with a report about the participant's glycemic control.

4.8 Detailed description of the data collection instrument

These activities will be performed by the partner startup (Klivo):
The intervention will begin with one 60-minute one-on-one orientation session about the program. The diabetes educator will call the participant at the previously scheduled time.

KIP will include tele-education via weekly phone calls over six weeks in one-on-one sessions about hypoglycemia, nutrition, physical activity, lifestyle, mental health, and regular use of medication as described below.

Sessions
1. Introduction. Questionnaires. One-on-one orientation about hypoglycemia.
2. Questionnaires. Problem-solving skills and IDEA approach (Identify the problem, Define possible solutions, Evaluate the solutions, Act on the best solution). Definition of an individualized plan for the subsequent interventional sessions.
3. One-on-one orientation about nutrition and physical activity.
4. One-on-one orientation about medications, especially about the relation between insulin administration time and meals.
5. One-on-one orientation about emotional health, smoking, and alcohol.
6. One-on-one orientation about healthy changes and glucose control in the long term.

Participants that require insulin therapy will take one more orientation session about administering the injection.

Monitoring
Nurses will make phone calls to the participants every fifteen days over twelve months to support ongoing self-management of T2DM. All participants will continue to receive treatment from their regular medical doctors during the 12-month time.

Moreover, depending on the seriousness of the situation, a phone call will be made, or a text message will be sent to the participants if abnormal parameters are detected. Phone calls will be made if capillary glucose is < 54 mg/dL or > 450 mg/dL, and text messages will be sent if capillary glucose is between 55 and 70 g/dL or 350 and 449 mg/dL. The participants will be monitored every 15 minutes to guarantee that hypo or hyperglycemia is reverted.

If specific needs are detected, nurses will suggest additional scheduled phone calls with a multidisciplinary team including psychologists, nutritionists, and physical educators.

Contacting medical doctors

If the participant presents three or more episodes of hypoglycemia, their medical doctor will receive an e-mail with an alert about their glycemic control.

Clinical and laboratorial measurements

Questionnaires

To obtain information related to the participants' demographic characteristics, medical history, and environmental risk factors, participants will answer questions during a phone call when the nurse will fill in an electronic questionary designed according to the ICHOM criteria [12]. Additionally, information about physical activity (time per week), smoking status, and alcohol consumption (amount and frequency per week) will be collected according to the ICHOM criteria [12].

To verify medication adherence, data will be collected via a standardized instrument called the Morisky Green scale [13]. The relationship between participants and T2DM will be evaluated by the Problem Areas in Diabetes (PAID) questionnaire [14]. The WHO Well-Being Index (WHO-5) instrument will be used to assess psychological well-being [15]. The depression status will be estimated by the Patient Health Questionnaire (PHQ-9) [16], and strategic directions will be suggested according to the score.

Blood pressure measurement

Systolic and diastolic blood pressures will be based on values reported by the participant during telephonic contact (self-reported).

Anthropometric parameters

Participants will report all the anthropometric parameters during telephonic contact. To measure waist circumference (WC), participants will be instructed to place a tape measure around their body, at the top of the hipbone, which is usually at the level of the umbilicus. Increased WC is defined as ≥ 88 cm for women and ≥ 102 cm for men.

Body mass index (BMI) will be calculated as the body weight (kg) divided by the squared height (m^2). Overweight will be defined as BMI ≥ 25 kg/m^2 and < 30 kg/m^2, and obesity will be defined as BMI ≥ 30 kg/m^2.

Biochemical analysis

Blood glucose, HbA1c, total cholesterol, triglycerides, and lipoprotein fractions as high-density lipoprotein (HDL-c) and low-density lipoprotein (LDL-c) will be measured by standard techniques in the participants' usual laboratory, after fasting for at least eight hours, every three months.

Disease diagnosis

Systolic blood pressure (SBP) ≥ 140 mmHg or diastolic blood pressure (DBP) ≥ 90 mmHg (measured in the doctor’s office or at home) or antihypertensive drug use will be used for hypertension diagnosis [18]. T2DM will be defined by the presence of fasting glucose ≥ 126 mg/dL or antidiabetic drug use. Dyslipidemia will be defined by drug use.

4.9 Statistical analysis

These activities will be performed by the investigator Prof. Dr. Camila Maciel de Oliveira and her advisee:
Analyses will be conducted by comparing pre- and post-intervention parameters of the patients selected for KIP.

For all the measurements, clinical and laboratorial characteristics will be assessed by descriptive statistics. Categorical variables will be expressed as percentages, and continuous variables will be expressed as mean ± SD or median (interquartile range). The Kolmogorov-Smirnov test will be used to check data normality. The characteristics of the participants in the different groups (defined according to clinical characteristics) will be evaluated by t-test or Wilcoxon rank test (continuous variables) or Pearson’s chi-squared test (categorical variables). Logistic regression will be used to assess the association between independent variables and diabetes mellitus. The analyses will be adjusted for age and sex. Receiver Operational Characteristics (ROC) curves will be performed to evaluate the performance of the suggested models and models that are scientifically sound, and the area under the curve (AUC) will be used to measure the discriminatory power of the identified explanatory variables for T2DM. Statistical analysis will be performed by using RStudio software version 1.3.1093. The significance level will be set at 5%.

Sub-groups (e.g., group I and group II) will be created on the basis of pre-existing conditions described as follows:

Group I: Participants with no or only mild non-proliferative diabetic retinopathy without any macular involvement; chronic kidney disease up to stage 3a (eGFR ≥ 45 mL/min/1.73 m^2); participants with no known macrovascular diseases.

Group II: Participants with retinal pathologies documented in the medical records, including proliferative diabetic retinopathy (moderate to severe) or other retinal or macular diseases; or chronic kidney disease stage 3b or 4; or known peripheral vascular, coronary, or cerebrovascular disease.

5. OUTCOMES
Expected academic results:

KIP participants should present improved HbA1c and TIR along the intervention as compared to baseline values.

6. RISKS AND BENEFITS

6.1 Risks: The program involves educational actions in health and monitoring (especially glycemic control). Risks are related to disease diagnosis (for example, hypo or hyperglicemia) and measures to mitigate such events. There are also risks involved with data handling (leak, hacker attack, risks to safety and privacy, for instance).

6.2 Paying for participants' expenses regarding the research:
Not applicable

6.3 Benefits:
Participants will receive information about the primary pathology (diabetes mellitus) and associated factors. Instant alerts will be sent to participant when abnormal glucose levels are detected.

7. TIME SCHEDULE

	2022										2023	
	M	A	M	J	J	A	S	O	N	D	J	F
Specification of objectives and hypotheses.												
Survey of the literature.												
Project approval by funding agencies												
Project approval by the Research Ethics Committee												
Operating concepts and variables.												
Data collection.												
Laboratory tests (if applicable)	NON-APPLICABLE)											
Data compilation.												
Results analysis.												
Statistical treatment and data analysis												
Discussion and conclusion												
Thesis defense/Presentation												
Publication in scientific journals.												

Data collection at baseline and the intervention will start on 03/03/2022. Data will be collected again 3, 6, 9, and 12 months after the start of the protocol.

8. NECESSARY RESOURCES AND PROJECT COSTS

8.1 Executing team:
Prof. Dr. Camila Maciel de Oliveira and Luiza Borcony Bolognesi

8.2 Equipment and supply:
Not applicable

8.3 Risks associated with the necessary resources:
Not applicable

8.4 Detailed budget:
The funding company will provide R$100,000.00 (~US$20,000.00) to fund the project

8.5 Sponsor: KLIVO LICENCIAMENTO LTDA (CNPJ (Corporate Taxpayer Number)15.996.337/0001-85) (See letter attached to the platform)

9. REFERENCES

[1] Hong JS, Wasden C, Han DH. Introdução da terapêutica digital. Programas de Métodos computacionais Biomed. 2021; 209: 106319. pmid: 34364181.

[2] Saeedi P, Petersohn I, Salpea P, Malanda B, Karuranga S, Unwin N, et al; Comitê atlas de diabetes do IDF. Estimativas de prevalência global e regional de diabetes para 2019 e projeções para 2030 e 2045: Resultados do Atlas da Federação Internacional de Diabetes, 9ª edição. Diabetes Res Clin Pract. 2019; 157: 107843. pmid: 31518657.

[3] Rhee SY, Kim C, Shin DW, Steinhubl SR. Presente e futuro da saúde digital em diabetes e doença metabólica. Diabetes Metab J. 2020; 44(6): 819-27. pmid: 33389956.

[4] Thorpe K, Toles A, Shah B, Schneider J, Bravata MD. Redução associada à perda de peso nos gastos com assistência médica para indivíduos com seguro comercial com condições crônicas. J Occup Environ Med. 2021; 16 de junho. doi: 10.1097/jom.0000000000002296. Epub à frente da impressão. pmid: 34138824.

[5] Huckfeld PJ, Frenier C, Pajewski NM, Espeland M, Peters A, Casanova R, et al. Associações de intervenção intensiva do estilo de vida no diabetes tipo 2 com uso de cuidados de saúde, gastos e incapacidade: estudo auxiliar do estudo look AHEAD. JAMA Netw Open. 2020; 3(11): e2025488. pmid: 33231638.

[6] Downing J, Bollyky J, Schneider J. Uso de um medidor de glicose conectado e treinamento certificado de educador de diabetes para diminuir a probabilidade de excursões anormais de glicose: o Programa Livongo para Diabetes. J Med Internet Res. 2017; 19(7): e234. pmid: 28698167.

[7] Bollyky JB, Bravata D, Yang J, Williamson M, Schneider J. Coaching de estilo de vida remoto, além de um medidor de glicose conectado com suporte certificado de educador de diabetes melhora a glicose e a perda de peso para pessoas com diabetes tipo 2. J Diabetes Res. 2018: 3961730. pmid: 29888288.

[8] Goh KLS, Lee CS, Koh CHG, Ling NL, Ang SB, Oh C. Avaliando a eficácia e a utilidade de um novo sistema de telemonitoramento culturalmente adaptado para melhorar o controle glicêmico dos asiáticos com diabetes mellitus tipo 2: um protocolo de estudo de método misto. Ensaios. 2021; 22(1): 305. pmid: 33902656.

[9] Porter ME, Teisberg EO. Redefinição da saúde - criando concorrência baseada em valor em resultados. Boston: Harvard Business School Press; 2006.

[10] Kaufman N, Khurana I. Utilizando tecnologia digital de saúde para prevenir e tratar o diabetes. Diabetes Technol Ther. 2016; 18 Suppl 1 (Suppl 1): S56-S68. pmid: 26836430.

[11] Beck J, Greenwood DA, Blanton L, Bollinger ST, Butcher MK, Condon JE, et al. 2017 National standards for diabetes self-management education and support. Diabetes Care. 2017; 40(10):1409-19. https://doi.org/10.2337/dci17-0025.

[12] Nano J, Carinci F, Okunade O, Whittaker S, Walbaum M, Barnard-Kelly, K, et al.; Grupo de Trabalho de Diabetes do Consórcio Internacional de Medição de Resultados em Saúde (ICHOM). Um conjunto padrão de desfechos centrados nas pessoas para diabetes

mellitus: resultados de uma abordagem internacional e unificada. Diabet Med. 2020; 37(12): 2009-18. pmid: 3214488.

[13] Morisky DE, Green LW, Levine DM. Concurrent and predictive validity of a self-reported measure of medication adherence. Med Care. 1986; 24(1): 67-74. pmid: 3945130.
[14] Schmitt A, Reimer A, Kulzer B, Haak T, Ehrmann D, Hermanns N. How to assess diabetes distress: comparison of the Problem Areas in Diabetes Scale (PAID) and the Diabetes Distress Scale (DDS). Diabet Med. 2016; 33(6): 835-43. pmid:26287511.
[15] Topp CW, Østergaard SD, Søndergaard S, Bech P. The WHO-5 Well-Being Index: a systematic review of the literature. Psychother Psychosom. 2015; 84(3): 167-76. pmid: 25831962.
[16] Koenke K, Spitzer RL, Williams JBW. The PHQ-9 validity of a brief depression severity measure. J Gen Intern Med. 2001; 16(9): 606-13. pmid: 11556941.
[17] Munshi MN, Meneilly GS, Rodríguez-Mañas L, Close KL, Conlin PR, Cukierman-Yaffe T, et al. Diabetes in ageing: pathways for developing the evidence base for clinical guidance. Lancet Diabetes Endocrinol. 2020; 8(10): 855-67. pmid: 32946822.
[18] Barroso WKS, Rodrigues CIS, Bortolotto LA, Mota-Gomes MA, Brandão AA, Feitosa ADM, et al. Diretrizes Brasileiras de Hipertensão Arterial – 2020. Arq. Bras. Cardiol. 2021; 116(3): 516-658. https://doi.org/10.36660/abc.20201238

THE PROJECT WAS APPROVED BY THE RESEARCH ETHICS COMMITEE, ON THE BASIS OF THE MODEL OF THE FREE INFORMED CONSENT/ QUESTIONNAIRES AND/OR DATA COLLECTION INSTRUMENT

Nothing else was included in the above-mentioned document. Therefore, I return it with this translation in 17 pages, in accordance with my best understanding of its contents, which I have proofread.

On 09 March 2022
