# Supplementary material for: A data-driven approach to manage type 2 diabetes mellitus through digital health: The Klivo Intervention Program protocol (KIPDM)
Source: PLoS One. 2023 Feb 24;18(2):e0281844. doi: 10.1371/journal.pone.0281844 (PMC9956061; doi:10.1371/journal.pone.0281844)
Supplement: S1 File — (PDF) [file pone.0281844.s004.pdf]

PARECER CONSUBSTANCIADO DO CEP

DADOS DO PROJETO DE PESQUISA

Título da Pesquisa: Protocolo do Programa de Intervenção Klivo: gerenciamento do diabetes mellitus tipo 2 através de uma plataforma digital

Pesquisador: Camila Maciel de Oliveira

Área Temática:

Versão: 2

CAAE: 53899421.1.0000.5137

Instituição Proponente: Sociedade Mineira de Cultura

Patrocinador Principal: KLIVO LICENCIAMENTO LTDA.

DADOS DO PARECER

Número do Parecer: 5.246.322

Apresentação do Projeto:

Programas remotos específicos ajudam a prevenir, gerenciar ou tratar doenças crônicas. Essas intervenções têm desempenhado um papel essencial na gestão de condições metabólicas, como o diabetes mellitus tipo 2, que tem alto impacto social e econômico. Este estudo propõe avaliar uma estratégia de saúde digital em fase inicial de implementação. O Programa de Intervenção Klivo é um método intensivo de intervenção de estilo de vida que busca gerenciar o estilo de vida diário de adultos com diabetes mellitus tipo 2, com idade > 18 anos, com hemoglobina glicada (HbA1c) de 7% ou mais. O programa é gratuito para os participantes inscritos em planos de saúde e organizações prestadoras de plataformas de saúde parceiras. Os resultados como HbA1c e tempo na faixa de glicemia-alvo serão avaliados no momento que antecede a fase de intervenção e nos pontos de tempo estipulados. O programa será baseado em um processo de gestão de 12 meses durante o qual os indivíduos serão supervisionados remotamente por enfermeiros a cada 15 dias. Quando forem detectados parâmetros anormais de glicemia, indivíduos e médicos responsáveis serão contatados de acordo com o protocolo proposto. Os dados clínicos e laboratoriais, controle de peso, qualidade de vida, saúde mental, adesão a medicamentos, confiança na autogestão, utilização da assistência à saúde, alfabetização diabética e sofrimento relacionado à doença serão avaliados por meio de questionários eletrônicos validados. O programa incluirá tele-educação através de telefonemas semanais ao longo de seis semanas. Os exames laboratoriais e dados reportados ao telefone serão avaliados na linha de base e 3, 6, 9 e 12 meses após a intervenção,

Endereço: Av. Dom José Gaspar, 500 - Prédio 03, sala 228
Bairro: Coração Eucarístico **CEP:** 30.535-901
UF: MG **Município:** BELO HORIZONTE
Telefone: (31)3319-4517 **Fax:** (31)3319-4517 **E-mail:** cep.proppg@pucminas.br

sendo os questionários aplicados no primeiro e último meses. Os achados deste estudo fornecerão uma visão sobre a melhora da saúde de indivíduos com diabetes mellitus tipo 2 e possivelmente indivíduos com outras doenças cardiometabólicas, incluindo hipertensão arterial, dislipidemia e obesidade.

Objetivo da Pesquisa:

Objetivos Primários:

1. Comparar os valores de HbA1c do indivíduo na linha de base e 3, 6, 9 e 12 meses após a inclusão no PIK.
2. Identificar a porcentagem de Tempo na Faixa (TIR) e, consequentemente, o número de eventos hipoglicêmicos graves ao longo de 12 meses.

Objetivos Secundários:

- Avaliação da incidência de complicações secundárias como lesões em retina, renal, cardíaca e cerebrovascular nos meses 1 e 12.

Avaliação dos Riscos e Benefícios:

Riscos: O programa envolve ações educativas em saúde e monitoramento (especialmente controle glicêmico). Há riscos relacionados ao diagnóstico da doença (hipoglicemia ou hiperglicemia, por exemplo) e as medidas tomadas para mitigar tais eventos. Há também riscos envolvendo manuseio de dados (vazamento de dados, ataques hackers, riscos à segurança e privacidade, por exemplo). Conforme descrito no TCLE, em caso de idenficação de situação de hipo ou hiperglicemia, a seguinte medida será tomada: "...seu médico receberá um e-mail com um relatório sobre o seu controle glicêmico."

Benefícios: Os pacientes receberão informações acerca da patologia de base (diabetes mellitus) e fatores associados. Alertas imediatos serão enviados aos pacientes, quando valores glicêmicos forem anormais.

Comentários e Considerações sobre a Pesquisa:

O projeto é relevante e exequível. Atende aos requisitos éticos da pesquisa envolvendo seres humanos.

Considerações sobre os Termos de apresentação obrigatória:

Os termos de apresentação obrigatória foram anexados e estão de acordo com as normas vigentes.

Endereço: Av. Dom José Gaspar, 500 - Prédio 03, sala 228
Bairro: Coração Eucarístico **CEP:** 30.535-901
UF: MG **Município:** BELO HORIZONTE
Telefone: (31)3319-4517 **Fax:** (31)3319-4517 **E-mail:** cep.proppg@pucminas.br

Conclusões ou Pendências e Lista de Inadequações:

Considerando o exposto e tendo em vista as Resoluções que norteiam a pesquisa envolvendo Seres Humanos consideramos o protocolo de pesquisa SEM PENDÊNCIAS, devendo o pesquisador acatar as orientações conforme o disposto no Parecer Consubstanciado.

Considerações Finais a critério do CEP:

Este parecer foi elaborado baseado nos documentos abaixo relacionados:

Tipo Documento	Arquivo	Postagem	Autor	Situação
Informações Básicas do Projeto	PB_INFORMAÇÕES_BÁSICAS_DO_PROJETO_1830509.pdf	09/02/2022 17:16:39		Aceito
Projeto Detalhado / Brochura Investigador	Projeto_Detalhado_PARA_PUC_MINAS_versao_2.doc	09/02/2022 17:12:50	Camila Maciel de Oliveira	Aceito
Outros	CartaRespostaaoParecerConsubstanciadodatadode30dedezembrode2021.docx	09/02/2022 17:11:48	Camila Maciel de Oliveira	Aceito
Outros	ModelosdeQuestionarios.pdf	09/02/2022 16:53:51	Camila Maciel de Oliveira	Aceito
TCLE / Termos de Assentimento / Justificativa de Ausência	TCLE.pdf	09/02/2022 16:42:34	Camila Maciel de Oliveira	Aceito
Outros	TAI.pdf	09/02/2022 16:38:15	Camila Maciel de Oliveira	Aceito
Outros	TCUD.pdf	31/01/2022 09:59:00	Camila Maciel de Oliveira	Aceito
Declaração de Instituição e Infraestrutura	KlivoDeclaracaodeInstituicaoPUCMinas.docx	30/11/2021 10:16:06	Camila Maciel de Oliveira	Aceito
Declaração do Patrocinador	Klivo_Declaracao_de_Patrocinador_PUC_Minas.docx	30/11/2021 10:12:50	Camila Maciel de Oliveira	Aceito
Projeto Detalhado / Brochura Investigador	Projeto_Detalhado_PARA_PUC_MINAS.doc	30/11/2021 10:11:49	Camila Maciel de Oliveira	Aceito
Folha de Rosto	Folha_de_Rosto.pdf	01/10/2021 11:20:55	Camila Maciel de Oliveira	Aceito

Situação do Parecer:

Aprovado

Necessita Apreciação da CONEP:

Não

BELO HORIZONTE, 16 de Fevereiro de 2022

Assinado por:
CRISTIANA LEITE CARVALHO
(Coordenador(a))
