# Supplementary material for: A data-driven approach to manage type 2 diabetes mellitus through digital health: The Klivo Intervention Program protocol (KIPDM)
Source: PLoS One. 2023 Feb 24;18(2):e0281844. doi: 10.1371/journal.pone.0281844 (PMC9956061; doi:10.1371/journal.pone.0281844)
Supplement: S2 File — (PDF) [file pone.0281844.s005.pdf]

CYNTHIA MARIA DE CAMPOS PRADO MANSO
TRADUTORA PÚBLICA E INTÉRPRETE COMERCIAL – INGLÊS
Matrícula JUCESP nº 792
C.C.M. 9821401 RG 20.907.343-3-SSP-SP CPF 180.988.858-10
Rua Campos Salles nº 1150 apto. 11 – Centro – CEP 14015-110
RIBEIRÃO PRETO – SP – TEL (16) 3630-8603 – CEL (16) 99993-3932

To whom it may concern, I declare that I am a Sworn Translator and Commercial Interpreter duly sworn by the Board of Trade of the State of São Paulo (JUCESP) - Federative Republic of Brazil and I certify in good faith that I am fluent in both the English and Portuguese languages; that on this date an original document in the Portuguese language identified as PONTIFÍCIA UNIVERSIDADE CATÓLICA DE MINAS GERAIS PUCMG – PARECER CONSUBSTANCIADO DO CEP DADOS DO PROJETO DE PESQUISA TÍTULO DO PROJETO PROTOCOLO DO PROGRAMA DE INTERVENÇÃO KLIVO: GERENCIAMENTO DO DIABETES MELLITUS TIPO 2 ATRAVÉS DE UMA PLATAFORMA DIGITAL was presented to me by its bearer for the sole purpose of translating said document into English; and that the text attached below is a true and complete translation of said document to the best of my knowledge and ability:

[Logo on the top left of all document pages]: PUC Minas, The Pontifical Catholic University of Minas Gerais
[Logo on the top right of all document pages]: Plataforma Brasil, the Brazilian platform for submission of research projects to Research Ethics Committees

[Header on all the document pages]: THE PONTIFICAL CATHOLIC UNIVERSITY OF MINAS GERAIS – PUCMG

[Footer on all the document pages]: **Address:** Av. Dom José Gaspar, 500 – Prédio 03, sala 228
District: Coração Eucarístico **CEP:** 30.535-901
State: MG **City:** BELO HORIZONTE
Telephone and Fax number: (31)3319-4517
Email address: cep.proppg@pucminas.br

CONSOLIDATED OPINION OF THE RESEARCH ETHICS COMMITTEE (REC)

INFORMATION ABOUT THE RESEARCH PROJECT

Title of the Research: The Klivo Intervention Program protocol: management of type 2 diabetes mellitus through a digital platform
Researcher: Camila Maciel de Oliveira
Theme Area:
Version: 2
Certificate of Ethics Appreciation Presentation: 53899421.1.0000.5137
(CAAE, abbreviation in Portuguese)
Proponent Institution: Sociedade Mineira de Cultura
Main Sponsor: KLIVO LICENCIAMENTO LTDA.

INFORMATION ABOUT THE CONSOLIDATED OPINION

Consolidated Opinion Number: 5.246.322

Presentation of the Project:
Specific remote programs help prevent, manage, and treat chronic diseases. These interventions have played an essential role in the management of metabolic conditions, such as type 2 diabetes mellitus, which has high social and economic burden. This study proposes evaluating a digital health strategy in the initial phase of implementation. The Klivo Intervention Program is an intensive lifestyle intervention method that seeks to manage the daily lifestyle of adults with type 2 diabetes mellitus, aged > 18 years, with glycated hemoglobin (HbA1c) of 7% or higher. The program is free for patients that are users of partner health plans and healthcare provider organizations. Results like HbA1c and time in target blood glucose range will be evaluated at baseline and stipulated time points. The program will be based on a 12-month management process during which participants will be remotely supervised by nurses every 15 days. When abnormal glucose levels are detected, the participant and their medical doctor will be contacted according to an established protocol. Clinical and laboratory data, weight control, quality of life, mental health, medication adherence, confidence in self-management, healthcare utilization, diabetic literacy and related distress will be evaluated through validated electronic questionnaires. The program will include tele-education via phone calls along the first six weeks. The laboratorial data and data reported over the phone will be assessed at baseline and 3, 6, 9, and 12 months after intervention;

the questionnaires will be applied in the first and last months. Findings from this study will provide insights into the health improvement of individuals with type 2 diabetes mellitus and possibly of individuals with other cardiometabolic conditions, including hypertension, dyslipidemia, and obesity.

Objectives of the Research

Primary Objectives:

1. To compare the participant’s HbA1c values at baseline and 3, 6, 9, and 12 months after inclusion in KIP.
2. To identify the percentage of time in range (TIR) and, hence, the number of severe hypoglycemic events over 12 months.

Secondary objectives:

To evaluate the incidence of secondary complications like retinal, renal, cardiac, and cerebrovascular lesions at months 1 and 12.

Evaluation of Risks and Benefits

Risks: The program involves educational actions in health and monitoring (especially glycemic control). Risks are related to disease diagnosis (for example, hypo or hyperglicemia) and measures to mitigate such events. There are also risks involved with data handling (leak, hacker attack, risks to safety and privacy, for instance). As described in the Free Informed Consent, if a hypo or hyperglycemia event is identified, the following measure will be taken: “… your medical doctor will receive an email with a report about your glycemic control.”

Benefits: Participants will receive information about the primary pathology (diabetes mellitus) and associated factors. Instant alerts will be sent to participant when abnormal glucose levels are detected.

Comments and Considerations about the Research:

The project is relevant and feasible. It meets the ethical requirements for research involving humans.

Considerations about the Terms that must be presented

The terms have been attached to the platform and abide by the current norms.

Conclusions or Pending Matters or List of Inadequacies
From what has been presented and bearing in mind the Resolutions that guide research involving Humas, we consider that the project has NO PENDING MATTERS, and that the researcher must abide by the guidelines listed in the Consolidated Opinion.

Final Considerations by the REC:

This consolidated opinion was drafted on the basis of the following documents:

Document Type	File	Uploaded on	Author	Status
Basic Information about the Project	PB_INFORMAÇÕES_BÁSICAS_DO_PROJETO_1830509.pdf	09/Feb/2022 17:16:39		Accepted
Detailed Project	Projeto_Detalhado_PARA_PUC_MINAS_versao_2.doc	09/Feb/2022 17:12:50	Camila Maciel de Oliveira	Accepted
Others	CartaRespostaaoParecerConsubstanciadode30dedezembrode2021.docx	09/Feb/2022 17:11:48	Camila Maciel de Oliveira	Accepted
Others	ModelosdeQuestionarios.pdf	09/Feb/2022 16:53:51	Camila Maciel de Oliveira	Accepted
Free Informed Consent	TCLE.pdf	09/Feb/2022 16:42:34	Camila Maciel de Oliveira	Accepted
Others	TAI.pdf	09/Feb/2022 16:38:15	Camila Maciel de Oliveira	Accepted
Others	TCUD.pdf	31/Jan/2022 09:59:00	Camila Maciel de Oliveira	Accepted
Statement of Institution and Infrastructure	KlivoDeclaracaodeInstituicaoPUCMinas.docx	30/Nov/2021 10:16:06	Camila Maciel de Oliveira	Accepted
Sponsor’s Statement	Klivo_Declaracao_de_Patrocinador_PUC_Minas.docx	30/Nov/2021 10:12:50	Camila Maciel de Oliveira	Accepted
Detailed Project	Projeto_Detalhado_PARA_PUC_MINAS.doc	30/Nov/2021 10:11:49	Camila Maciel de Oliveira	Accepted
Cover Page	Folha_de_Rosto.pdf	01/Oct/2021 11:20:55	Camila Maciel de Oliveira	Accepted

Status of the Consolidated Opinion:
Approved

Requires Appreciation by the National Council of Research Ethics Committees:

No

BELO HORIZONTE, 16 February 2022
Signed by CRISTIANA LEITE CARVALHO (Coordinator)

Nothing else was included in the above-mentioned document. Therefore, I return it with this translation in 17 pages, in accordance with my best understanding of its contents, which I have proofread and thus sign.

On 09 March 2022
