# Supplementary material for: A data-driven approach to manage type 2 diabetes mellitus through digital health: The Klivo Intervention Program protocol (KIPDM)
Source: PLoS One. 2023 Feb 24;18(2):e0281844. doi: 10.1371/journal.pone.0281844 (PMC9956061; doi:10.1371/journal.pone.0281844)
Supplement: S3 File — (PDF) [file pone.0281844.s006.pdf]

São Paulo, April 06, 2022

TO WHOM IT MAY CONCERN

I, André Soares Sá, Manager of Klivo Licenciamento LTDA, located at Rua Afonso Braz, 373, Vila Nova Conceição, São Paulo – SP, Brazil, declare that Klivo Licenciamento LTDA has funded the project titled **A data-driven approach to manage type 2 diabetes mellitus through digital health: The Klivo Intervention Program protocol**, authored by Camila Maciel de Oliveira, Luiza Borcony Bolognese, Mercedes Bacells, Chunyu Liu, and Clemente Nobrega.

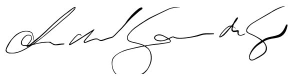

Andre Soares Sá
Manager
Klivo Licenciamento LTDA

KLIVO LICENCIAMENTO LTDA
CNPJ 35.996.337/0001-85
Rua Afonso Braz, 373 - Vila Nova Conceição
São Paulo - SP, Brazil
