# Supplementary material for: A data-driven approach to manage type 2 diabetes mellitus through digital health: The Klivo Intervention Program protocol (KIPDM)
Source: PLoS One. 2023 Feb 24;18(2):e0281844. doi: 10.1371/journal.pone.0281844 (PMC9956061; doi:10.1371/journal.pone.0281844)
Supplement: S4 File — (PDF) [file pone.0281844.s007.pdf]

São Paulo, January 11th, 2023

TO WHOM IT MAY CONCERN

I, André Soares Sá, Manager of Klivo Licenciamento LTDA, located at Rua Afonso Braz, 373, Vila Nova Conceição, São Paulo – SP, Brazil, declare that Klivo Licenciamento LTDA has funded the project titled **A data-driven approach to manage type 2 diabetes mellitus through digital health: The Klivo Intervention Program protocol**, authored by Camila Maciel de Oliveira, Luiza Borcony Bolognese, Mercedes Bacells, Chunyu Liu, and Clemente Nobrega.

I declare that the protocol has permission to be shared publicly and published. I am aware that the PLOS Editorial Policy requires that the original protocol be published alongside the manuscript in case you accept it. Also, in case the paper is accepted, all content, including the protocol, will be published under the Creative Commons Attribution (CC BY) 4.0 license, which means that it will be freely available online, and any third party is permitted to access, download, copy, distribute, and use these materials in any way, even commercially, with proper attribution.

I declare the research team has permission to share this document to publish this protocol under CC BY 4.0 if the work is accepted.

I affirm that the research team will be able to comply with this policy.

Andre Soares Sá Manager
Klivo Licenciamento LTDA

KLIVO LICENCIAMENTO LTDA CNPJ 35.996.337/0001-85
Rua Afonso Braz, 373 - Vila Nova Conceição São Paulo - SP, Brazil
